# Optimizing working memory assessment: development of shortened versions of complex spans, updating, and binding tasks

**DOI:** 10.1007/s00426-025-02083-7

**Published:** 2025-03-08

**Authors:** Fábio Monteiro, Letícia Botan Nascimento, José Augusto Leitão, Eduardo J. R. Santos, Paulo Rodrigues, Isabel M. Santos, Fátima Simões, Carla S. Nascimento

**Affiliations:** 1 CINEICC - Center for Research in Neuropsychology and Cognitive Behavioral Intervention, Faculty of Psychology and Educational Sciences, University of Coimbra, Coimbra, Portugal; 2 Chronocog – Laboratory for Chronopsychology and Cognitive Systems, Faculty of Psychology and Educational Sciences, University of Coimbra, Coimbra, Portugal; 3 Department of Psychology and Education, University of Beira Interior, Covilhã, Portugal; 4 William James Center for Research, University of Aveiro, Aveiro, Portugal; 5 Faculty of Psychology and Educational Sciences, University of Coimbra, Rua do Colégio Novo, 3000-115 Coimbra, Portugal; 6 SHERU – Sport, Health & Exercise Research Unit, Polytechnic Institute of Castelo Branco, Castelo Branco, Portugal; 7 Center for Research in Education and Psychology, University of éVora, Évora, Portugal

## Abstract

Given the lengthy administration of most working memory (WM) tasks, some researchers have developed reduced versions of these tests. However, they have focused primarily on complex spans. Recent studies suggested that estimating working memory capacity (WMC) using multiple tasks from different paradigms enhances measurement accuracy by isolating WMC variation from task- and paradigm-specific influences. Considering this, we evaluated whether complex spans, updating, and binding tasks could be shortened while maintaining robust psychometric properties. Participants completed full-length versions of tests from these paradigms, which were then segmented into early, intermediate, and later trial blocks. The shortened WM tasks were based on the early trial blocks. They accounted for most of the variance in a set of factor scores derived from the full-length versions of the WM tests (*R*^*2*^ = 0.90). Additionally, the shortened and full-length versions presented a similar ability to predict fluid intelligence (*Gf*). The shortened tasks reduced administration time by 35%, saving around 30 min. To help researchers select the most suitable combination of shortened and/or full-length tasks, we calculated the *Gf* and WMC variance predicted by every possible task combination and the respective administration time. We believe that the shortened WM tasks will be highly valuable to researchers, as they provide reliable and valid WMC estimates in a time-efficient manner. We also examined whether using tests from different paradigms provides better WMC estimates than employing collections of tasks from the same class. Our results confirmed this hypothesis, highlighting the importance of diverse task selection to accurately assess WMC.

## Introduction

Working memory (WM) is a system with limited capacity that temporarily stores, manipulates, and retrieves information necessary for ongoing cognitive processes (Baddeley, 2012; Unsworth et al., 2009a). Individual differences in working memory capacity (WMC) are major predictors of high-order capacities, such as fluid intelligence (*Gf*) (Felez-Nobrega et al., 2018; Rey-Mermet et al., 2019) rationality (Burgoyne et al., 2023), and problem-solving (Friedman & Miyake, 2004). Additionally, WMC influences real-world behaviours, like multitasking (Colom et al., 2010) and emotion regulation (Barkus, 2020). Given the central role of WM in cognition, assessing this construct is ubiquitous in experimental and cognitive psychology (Conway et al., 2005). However, WM is also often measured in clinical, educational, and social investigations (Allen et al., 2020; Li et al., 2019; Mazerolle et al., 2012).

Multiple classes of cognitive tasks are used to measure WMC. Among them, the complex span is the most prevalent (Conway et al., 2005; Redick et al., 2012). However, there has been a recent increase in the use of binding and updating tasks (Bartsch et al., 2018; Waris et al., 2017; Wilhelm et al., 2013). Complex spans are dual tasks where participants must remember a series of stimuli presented in quick succession while performing a secondary task (Redick et al., 2012; Unsworth et al., 2009a, b). Updating tasks require participants to continuously refresh their mental representations (Ecker et al., 2010). Binding tasks involve combining different aspects of stimuli, such as their position and verbal content, to build new structures and associations (Oberauer et al., 2003). Although these task paradigms have different structures and features, they measure the same underlying construct — they loaded on the same latent factor in multiple studies (Lewandowsky et al., 2010; Schmiedek et al., 2009, 2014; Waris et al., 2017; Wilhelm et al., 2013). Moreover, they all serve as good predictors of *Gf* (Engle et al., 1999; Gray et al., 2017; Shelton et al., 2009).

However, most WM tasks take a long time to administer (Foster et al., 2015; Gonthier et al., 2016). For instance, completing a single complex span can take around 20 min (Unsworth et al., 2005). Many updating and binding tasks require approximately the same amount of time (Stollery & Christian, 2016; Waris et al., 2021), although some tests within these paradigms have shorter administration times (7 ~ 10 min) (Garcia et al., 2014; Kattner, 2021). These lengthy procedures can lead to long and tedious experimental sessions, potentially decreasing participant motivation and negatively impacting performance (Heitz et al., 2008). Furthermore, researchers often need to assess multiple constructs within limited timeframes, making it challenging to include several measures to evaluate all areas of interest (Stanton et al., 2002).

Balancing the limited amount of time to collect data and the extended administration times of most WM tasks has led many investigations to use a single test to measure WMC (Ma et al., 2017; Oswald et al., 2015). However, relying on a single task can result in biased interpretations, as no cognitive test is a perfect representation of the construct it is supposed to measure. Performance on these tasks is influenced not only by the construct of interest but also by specific features of the paradigm and task (e.g., the structure of the task or the content domain of the stimuli) and other forms of measurement error (Shipstead et al., 2012; Unsworth et al., 2009a). It is not possible to disentangle these sources of variance by applying a single task. Consequently, it becomes challenging to determine whether the individual differences detected by the test are mainly driven by the construct of interest or by unrelated sources of variation (Foster et al., 2015; Lewandowsky et al., 2010). Repeatedly using the same task in multiple studies can further confound these sources of variance, increasing the risk of interpreting task-specific variance as construct variance on a broader scale (Schmiedek et al., 2014).

To obtain a more accurate estimate of WMC, several authors recommended administering multiple WM tasks and either extracting the common variance at a latent level or computing a composite score by averaging the scores of all tests (Conway et al., 2005; Hicks et al., 2016). Estimates based on multiple tests from different paradigms and content domains (e.g., verbal, numeric, visuospatial) provide particularly accurate representations of WMC, as they allow the separation of construct-relevant variance from paradigm- and task-specific variance and measurement error (Gonthier et al., 2016; Waris et al., 2017). Additionally, using heterogeneous sets of valid measures that differ in construct-irrelevant features tends to lead to better estimates of the target construct than applying sets of similar tasks, as the former covers wider sources of relevant variance (Little et al., 1999). Schmiedek’s et al. (2014) suggested that this premise can be applied to WM tests. The outcomes of this investigation revealed that triplets of tasks from different classes correlated more strongly with a *Gf* factor than trios of tests from the same paradigm (e.g., updating tasks). However, given the prominence of the complex span in WM literature, a large number of studies used multiple versions of this paradigm to assess WMC (Engle et al., 1999; Kane et al., 2004; Unsworth et al., 2009b).

Applying multiple tests to assess WMC naturally increases the duration of data collection sessions (Felez-Nobrega et al., 2018). To address this issue, some researchers have developed and validated shortened versions of WM tasks. For example, Foster et al. (2015) divided three complex spans (reading, symmetry, and rotation spans) into blocks representing early, intermediate, and later trials. They then assessed the ability of different combinations of blocks and tasks to predict a set of *Gf* factor scores. Their findings indicated that the early trial blocks alone were sufficient to obtain a valid and reliable estimate of WMC — the first third of trials from the three complex spans was still able to account for 90% of the *Gf* explained by the full-length versions of these tasks. Moreover, these shortened versions of the three tasks produced a better WMC estimate than the complete version of any complex span alone. The shortened complex spans took approximately 43 min to complete, compared to 62 min for the full-length versions. In their turn, Oswald et al. (2015) created reduced versions of the reading, operation, and symmetry spans by removing all blocks of trials with the largest and smallest set sizes and the third block from all set sizes. These versions required 15–25 min less to administer than the original versions, which took approximately 39 min to complete. At last, Gonthier et al. (2016) developed a Composite Complex Span which also included shortened versions of the reading, operation, and symmetry spans. These reduced versions included one block of trials for the lowest and highest set sizes and two blocks of trials for all other set sizes. In general, these three sets of shortened WM tasks demonstrated strong internal and criterion validity, high internal consistency, and temporal stability, while reducing testing time by approximately 31–38%.

However, these studies have focused exclusively on shortening tasks within the complex span paradigm. Considering the advantages of deriving WMC estimates from different classes of tests and the limited amount of time researchers often have for data collection, creating shortened versions of WM tasks from different paradigms could have major benefits. These reduced versions would allow researchers to obtain reliable WMC estimates, free from paradigm- and task-variance, at a reduced time cost.

In this study, we employed a methodology similar to that of Foster et al. (2015) to evaluate whether WM tasks from different paradigms (complex span, updating tasks, binding tasks) could be shortened while maintaining good psychometric properties (internal consistency, and internal and criterion validity). To test this hypothesis, our participants completed the versions of the reading span, operation span, symmetry span, n-back task, working memory updating task, and binding and maintenance task included in the OpenWMB (Monteiro et al., 2024) as well as three reasoning tests. The OpenWMB is an open-source and automated battery containing multiple complex spans, updating, and binding tasks. The battery can be freely downloaded from the GitHub repository associated with the webpage 10.5281/zenodo.10600494 — to access this repository, locate and click on the GitHub URL presented on the Zenodo page. The WM tests were shortened through an iterative process, whose goal was to reduce each task to the minimum number of trials needed to achieve an α value ≥ 0.70 (Adadan & Savasci, 2012; McDonald, 1999). The remaining trials from each task were grouped into equal-sized blocks representing intermediate and later trials. To determine if shortening the WM tasks affected their internal and criterion validity, we compared the percentage of variance in a set of WMC and *Gf* factor scores explained by the reduced version of the WM tasks (which corresponded to the early blocks of trials from the complete WM tests) to that explained by the versions of the tasks that also included the intermediate and later blocks of trials.

Additionally, we sought to assist researchers in selecting the most suitable combinations of tasks and trial blocks in future studies. Our goal was to identify the configurations that maximized the information extracted from WM tasks while minimizing administration time. To this end, we performed a permutation analysis in which we calculated and compared the amount of *Gf* and WMC variance predicted by every possible combination of blocks (early, early + intermediate, and early + intermediate + later) and tasks (reading span, operation span, symmetry span, n-back task, working memory updating task, and biding task) and the time required to administer each combination.

Finally, we examined whether the findings of Schmiedek et al. (2014), which suggested that administering multiple WM tasks from different paradigms provided better WMC estimates than only applying tasks from the same class of tests, were replicated with our sample. We analyzed whether sets of tasks and blocks that only included tests from different paradigms had a better capacity to predict a set of *Gf* factor scores than combinations exclusively comprised of tasks from the same category.

## Method

### Participants

A total of 169 individuals participated in this experiment. Participants were recruited via email or personal invitations (personal referrals and direct approaches on campuses). No payment or other form of compensation was granted for participation. Eligibility was restricted to Portuguese citizens aged 18 to 35 who held at least a high school diploma. Data from five participants were discarded because they did not meet the inclusion criteria: three did not possess Portuguese citizenship, and two were older than 35 years. Another participant was excluded for scoring zero on more than one cognitive test. Consequently, the analyses presented in this paper were based on data from 163 participants (53 male; age range = 18–33 years, mean age = 22.29, *SD* = 4.13). This sample comprised 110 university students and 53 non-student community members. Within the latter group, 15 participants possessed a high school diploma, while 38 individuals completed at least one higher education degree.

### Apparatus and procedure

Participants completed all tests included in the OpenWMB (reading span, operation span, symmetry span, n-back task, working memory updating task, binding and maintenance task, and the multimodal span^1^) and three measures of fluid intelligence (letter series, number series, and Raven’s Advanced Progressive Matrices). The OpenWMB was programmed in OpenSesame (version 3.3.11) (Mathôt et al., 2012) and uses the Mousetrap plugin (Kieslich & Henninger, 2017) to track mouse movements in the symmetry and multimodal spans.

The participants completed all cognitive tasks in a single session. Up to 12 participants were tested simultaneously in a soundproof room. Participants were given a 20-minute break midway through the session, during which they were offered snacks, including water, juice, fruit, cookies, and sandwiches. General instructions were provided by the research team at the start of the session. Specific instructions for each task were embedded in the program and were presented on the computer screen. Practice trials were completed before each task. The order of the tasks was counterbalanced between participants with a Latin square design (a form of partial counterbalancing) (Grant, 1948). The order of the tasks was controlled by an algorithm embedded in the OpenWMB which ensured that all tasks were presented an equal number of times in each position across participants.

## Materials

### Complex spans

*Reading span* (Daneman & Carpenter, 1980). During this task, participants were presented with multiple blocks^2^ of alternating sentences and letters. A sentence was displayed at the beginning of each block. Participants were required to read the sentence and determine if it contained a syntactic error (processing/distractor subtask). After the sentence, a single letter was depicted. Participants were asked to memorize the letter. The sequence-letter combination was presented between two and six times within each block. At the end of each block, participants were requested to recall the to-be-remembered letters in the order they were presented and type them in a response box. Block sizes ranged from two to six (total number of trials: 60) and were presented in ascending order. Three blocks were administered per set size. This task also included two practice blocks, each containing two sentences and two to-be-remembered letters. The time that each participant had to read a sentence and signal if it presented a syntactic error was adjusted individually — before completing the reading span, the participants read 20 sentences and evaluated whether they contained syntactic errors or not. The time that each participant had to process a sentence in the test blocks of the reading span was the same as his mean reaction time in the calibration task + 2.5 standard deviations (Unsworth et al., 2009a). This procedure, developed by Unsworth et al. (2005a), minimizes the opportunity to rehearse the to-be-remembered stimuli during the processing/distractor segment of the complex spans, which provides a more accurate estimate of the simultaneous storage and processing capacity of the participants (Redick et al., 2012).

**Fig. 1:**
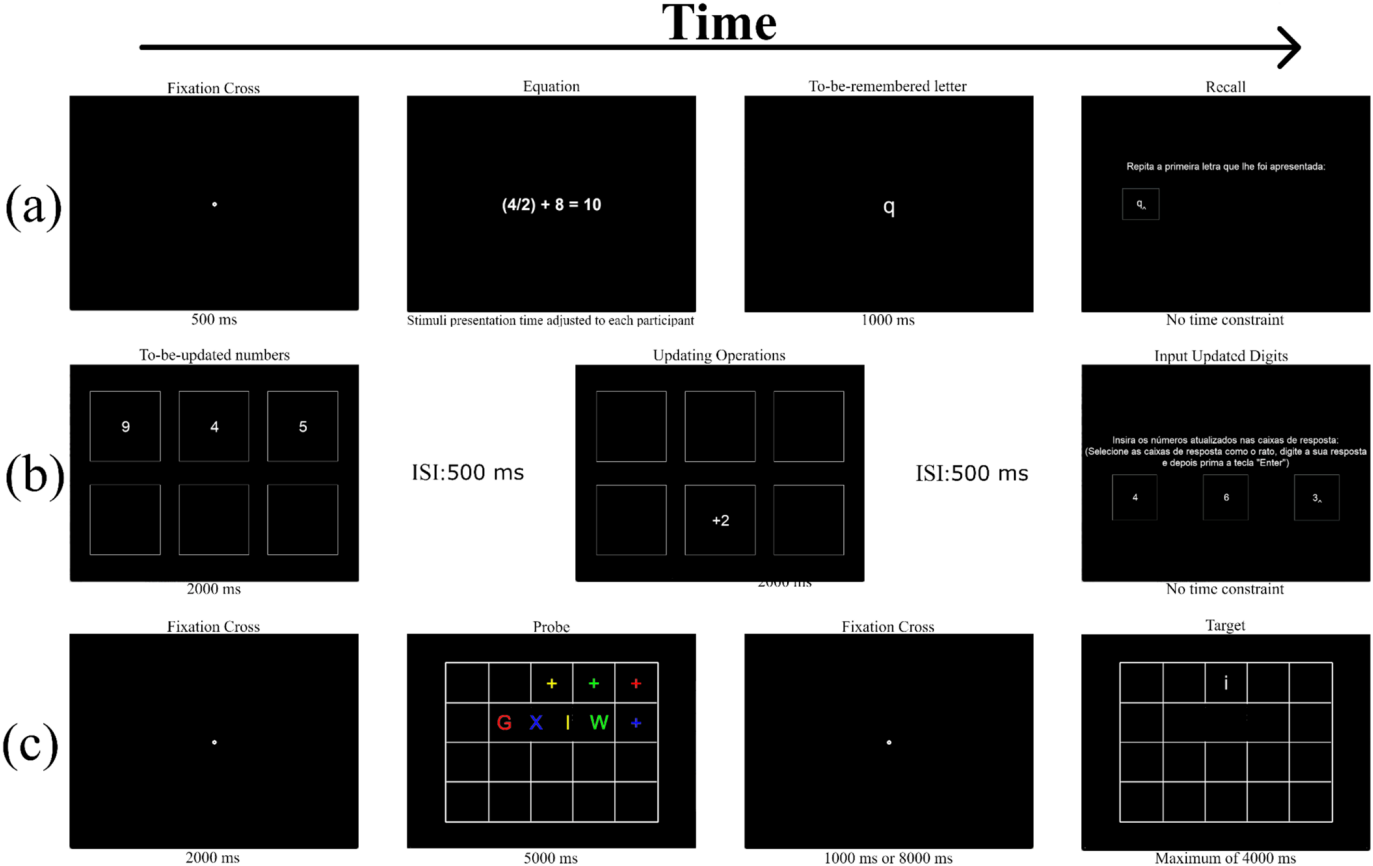
Diagram illustrating some of the tasks featured in the OpenWMB. **a** Operation span; **b** working memory updating task; **c** binding and maintenance task; ISI, interstimulus interval

*Operation span* (Turner & Engle, 1989). A schematic overview of the operation span is presented in Fig. 1a. This task followed the same procedure as the reading span, with one key difference: in the processing/distractor subtask, instead of reading sentences and identifying syntactic errors, the participants solved simple arithmetic equations (e.g., (5 * 2) + 4 = 14) and indicated whether the proposed result was correct. In trials in which the result suggested was incorrect, the difference between the proposed and correct solution was never higher than 2. After each equation, a single letter was displayed. At the end of each block, the participants had to recall the sequence of to-be-remembered letters and type it in a response box. Block sizes ranged from two to six (total number of trials: 60) and were presented in ascending order. Three blocks were administered per set size. This task also included two practice blocks, each containing two equations and two to-be-remembered letters. The time each participant had to complete the processing/distractor segment of each trial was adjusted individually by applying the same method used for the reading span. However, in this calibration task, the participants were asked to solve 20 equations (Unsworth et al., 2005).

*Symmetry span* (Kane et al., 2004). Unlike the previous two tasks, the symmetry span was exclusively comprised of visuospatial stimuli. In each trial, the participants first completed a processing/distractor subtask where they assessed whether an 8 × 8 matrix of black and white squares was symmetrical along its vertical axis or not (e.g., they had to decide whether the left half of the matrix mirrored the right half). In asymmetrical trials, a single square differed between the right and left halves of the matrix. After each matrix, a single red square was presented on a 4 × 4 grid. Participants were instructed to memorize the positions of these squares. At the end of each block of trials, participants recalled the sequence of red squares and typed their position on an empty 4 × 4 grid. Block sizes ranged from two to six (total number of trials: 60) and were presented in ascending order. Three blocks were administered per set size. This task also included two practice blocks, each containing two symmetrical/asymmetrical matrices and two to-be-remembered red squares. The time each participant had to determine if a matrix was symmetric or not was individually adjusted using the same method applied during the administration of the reading and the operation span. In this case, participants evaluated whether 20 matrices were symmetrical in the calibration task (Unsworth et al., 2009a).

### Updating tasks

*N-back task* (Kirchner, 1958; Schmiedek et al., 2009). A continuous series of letters was presented on a 3 × 3 grid. Participants were instructed to press the key ‘m’ whenever the displayed stimulus was identical to the stimulus presented two trials ago — the same letter was presented in the same cell of the grid. The n-back task consisted of a practice block and a test block. The practice block included eight trials, while the test block contained 38. However, the first two trials of each block were preparatory because they had no reference stimulus to be compared with. Thus, only six trials of the practice block and 36 trials of the test block were scored. In both cases, one-third of the trials were targets (trials where the current stimulus matched the one presented two trials ago). In the remaining two-thirds of trials, there was a mismatch between the position and/or the verbal content of the current stimulus and the stimulus presented two trials ago.

*Working memory updating task* (Salthouse et al., 1991; Schmiedek et al., 2009). A graphical overview of this task is shown in Fig. 1b. In this task, participants were required to continuously update three single-digit numbers presented at the beginning of each trial. These numbers were displayed for 2000 ms in three frames presented on the top half of the screen. Participants were asked to memorize these digits. Then, a continuous sequence of additions and subtractions (e.g., ‘+4’ or ‘−7’) appeared in three frames displayed on the bottom half of the screen. Participants had to update the initial numbers by applying these operations to the corresponding digit that was presented on the first row of frames and memorize the updated numbers. After all updating operations were presented, a single row with three empty frames was displayed in the middle of the screen. Participants were then instructed to type the updated digits into the corresponding frames. This task included a practice block and a test block. Participants had to complete two trials in the practice block and 12 trials in the test block.

### Binding task

*Binding and maintenance task* (Quinette et al., 2006). A schematic representation of the binding and maintenance task is presented in Fig. 1c. At the beginning of each trial, a 5 × 4 grid was displayed. The grid featured four colored uppercase letters (red, yellow, blue, and green) at its center, and four crosses with matching colors placed randomly in the remaining 17 squares. Participants were requested to associate each colored letter with the location of the cross with the same color. After this initial display, the grid was replaced by a white fixation dot. This fixation dot was presented for 1000 ms in half of the trials and 8000 ms in the other half. Then, a grid with a single white lowercase letter was displayed. Participants needed to determine if the position of the white lowercase letter matched the location of the cross that presented the same color as the corresponding uppercase letter in the first grid. The task included a practice block with four trials and a test block with 16 trials.

### Gf tasks

*Letter series* (Schrepp, 1999; Simon & Kotovsky, 1963). This task was used to assess verbal inductive reasoning. In each trial, participants were presented with a series of letters that followed an unstated logical pattern (e.g., ‘abmcdmefmghm’). Participants had to identify the logical pattern, guess the next three letters of the sequence (‘ijm’), and type them on a response box. This task included a practice block (containing two series) and a test block. In the test block, participants had five minutes to complete a maximum of 15 letter series. They could manage their time freely, spending as much time as they wished on each trial (within the five-minute limit). Participants were informed of the remaining time at the start of each trial. The letter series were presented in ascending order of difficulty.

*Number series* (Thurstone, 1938). The number series was administered to assess numerical inductive reasoning. In this task, participants were presented with a sequence of digits that followed a logical pattern (e.g., ‘3’, ‘10’, ’24’, ’45’, ’73’). They had to discern the rule governing the series, predict its next digit(s) (‘108’), and type their response on a response box. The number series consisted of a practice block, containing two trials, and a test block, with 15 sequences. Similar to the letter series task, participants had five minutes to complete as many problems as possible. They had the freedom to spend as long as they wanted on each trial, within this limit. Participants were notified of the time left at the beginning of each trial. The series were presented in ascending order of difficulty.

*Raven’s Advanced Progressive Matrices (RAPM)* (Raven et al., 1998). This task was used to evaluate figural inductive reasoning. In each trial, the participants assessed a pattern of black and white figures arranged on a 3 × 3 schema where the bottom-right figure was missing. The figures followed a relational pattern both horizontally (left to right) and vertically (top to bottom). Participants had to select the figure that completed the pattern from eight possible choices. The task included a practice block and a test block. Participants completed the first two even-numbered problems from set II of the RAPM in the practice block. In the test block, they attempted to solve the 18 odd-numbered problems from the same set. Participants were given 10 min to solve as many problems as possible. They could manage their time freely, dedicating as much time as they wished to each trial (within the 10-minute limit). Participants were informed of the remaining time at the start of each trial. Trials were presented in ascending order of difficulty.

### Trial scoring

The raw scores of all complex span tasks were calculated using partial-credit load scoring. These scores reflected the proportion of all correctly recalled stimuli in the memory portions of these tasks (Conway et al., 2005). We adopted partial-credit scoring because previous investigations have demonstrated that this method yields higher internal consistency than absolute scores — absolute scoring only considers blocks where all items are recalled in the correct order (Conway et al., 2005; Redick et al., 2012). In the working memory updating task, 1 point was awarded for each correctly updated digit. Thus, the possible score for each trial ranged from 0 to 3. The raw scores of the n-back task were calculated by summing the number of correct responses in target trials. The raw scores of the binding and maintenance task and the *Gf* tasks reflected the number of correct responses in these measures.

### Power analysis

Monte Carlo simulations (Wolf et al., 2013) were conducted to estimate the minimum sample size required to compute the structural equation model (SEM) used to assess if the WM and the *Gf* tasks loaded on distinct but related latent factors. The simulations were performed in RStudio (version 4.1.3) using the package “simsem” (version 0.5.16) (Pornprasertmanit et al., 2022). We followed the procedure outlined by Muthén and Muthén (2002) to run this analysis. The criteria used to determine if the chosen sample size was sufficient to achieve the desired statistical power and unbiased parameter estimates were as follows: relative bias and standard error bias ≤ 0.10 for all parameters, relative standard error bias ≤ 0.05 for parameters of interest, coverage between 0.91 and 0.98, and statistical power ≥ 0.80.

**Fig. 2:**
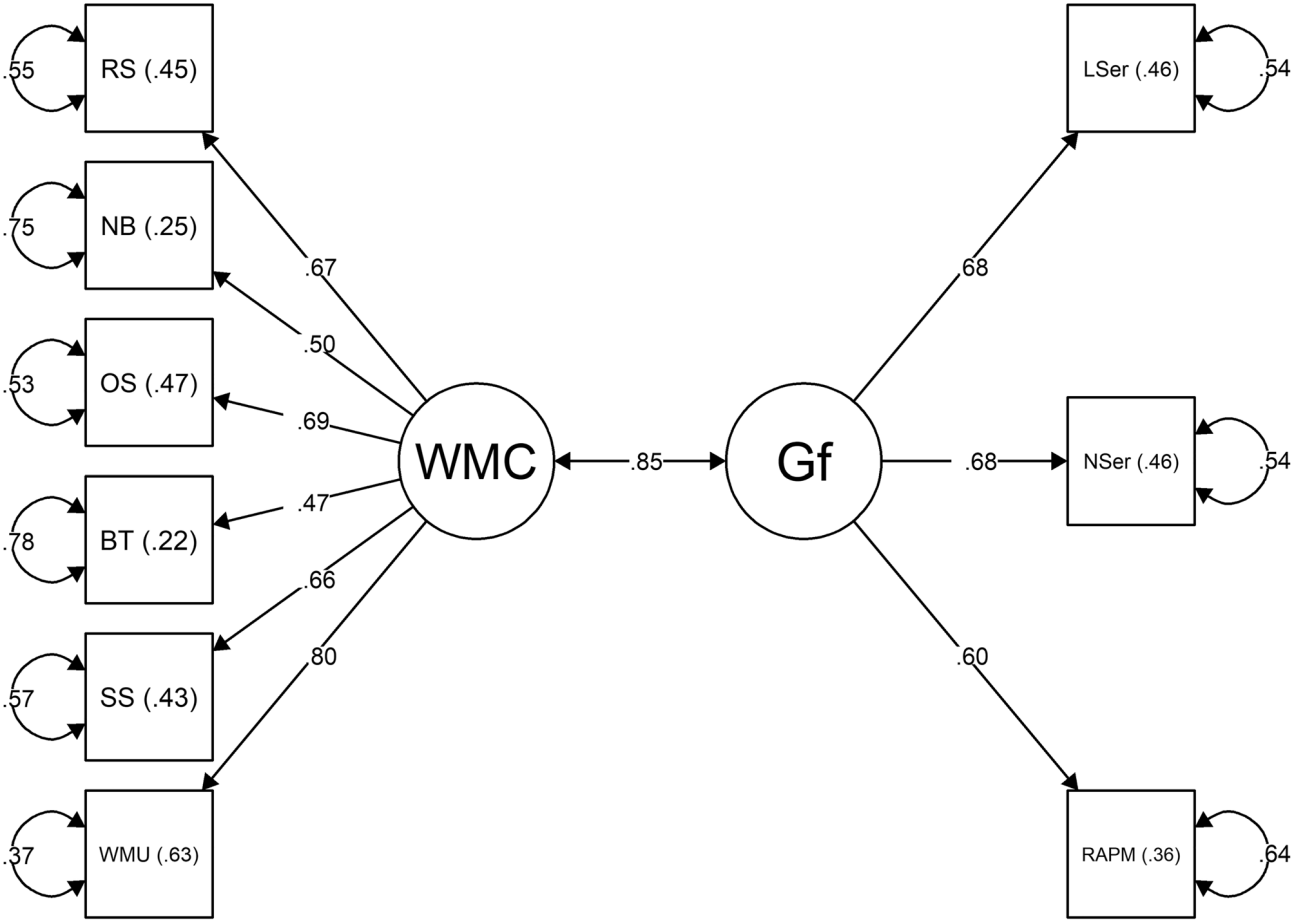
Model 1: *SEM* assessing the relationship between the *Gf* measures and the full-length versions of the WM tasks at a latent level. Circles represent latent factors. Rectangles represent manifest variables. Curved arrows represent standardized error terms. *RS* reading span, *OS* operation span, *SS* symmetry span, *NB* n-back task, *WMU* working memory updating task, *BT* binding and maintenance task, *WMC* working memory capacity, *Gf* fluid intelligence, *LSer* letter series, *NSer* number series, *RAPM* Raven’s Advanced Progressive Matrices

The parameter values used in the population model were sourced from previous investigations. The values for the reading span (0.70), operation span (0.66), n-back task (0.55), binding and maintenance task (0.86), letter series (0.71), and number series (0.70) were collected from the study of Wilhelm et al. (2013). The parameter value of the correlation between the WMC and *Gf* factors (0.83) was also derived from this study. Parameter values for the symmetry span (0.73) and the RAPM (0.76) were taken from the study of Kane et al. (2004), and the value for the working memory updating task (0.64) was obtained from the study of Schmiedek et al. (2009). After running the SEM, we recomputed the Monte Carlo simulation, replacing the values of the population model with the parameter estimates derived from the model we tested (Model 1, see Fig. 2). Both Monte Carlo simulations indicated that a minimum sample size of 130 was necessary to compute the proposed SEM with adequate statistical power and a reduced risk of committing a type I error (*α* = 0.05). Thus, these results suggested that the size of our sample was appropriate to implement the proposed analyses.

Several power analyses were conducted using G*Power (version 3.1.9.7) (Faul et al., 2009). An a priori power analysis was performed to determine the minimum sample size needed to compute the hierarchical linear regressions used to assess how well the shortened and full-length versions of the WM tasks predicted WMC and *Gf.* This analysis was based on the findings of Foster et al. (2015). These authors also split three complex spans into early, intermediate and later blocks of trials. Then, they employed hierarchical linear regressions to determine how much variance different combinations of these blocks could predict in two sets of WMC and *Gf* factor scores. The smallest effect size detected by Foster’s et al. (2015) (*f*^*2*^ = 0.47) was used for this calculation.

Using a *β* = 0.80 and an *α* = 0.05, the power analysis suggested that a minimum of 56 participants were required to detect an effect size of this magnitude with our most complex model. This model included 15 predictors, corresponding to all the early, intermediate, and latter blocks of trials from the six WM tasks. However, according to Cohen’s (1988) criteria, the effect size detected by Foster et al. (2015) was close to being considered large. To ensure the robustness of our results, we recalculated the analysis using a medium effect size (*f*^*2*^ = 0.15). This second analysis indicated that a sample size of 139 was necessary to run the linear regressions with acceptable statistical power. Thus, both power analyses suggested that the size of our sample (*N* = 163) was sufficient to conduct the aforementioned linear regressions and attain the desired power. This was confirmed by a *post hoc* power analysis that revealed that all regression models achieved a statistical power ≥ 0.98 (*α* = 0.05).

A final a priori power analysis was conducted to establish the minimum sample size needed to implement the one-tailed Mann-Whitney *U* test used to assess whether heterogeneous sets of WM tasks predict more *Gf* variance than their homogenous counterparts. To our knowledge, no prior study used a similar statistical procedure to evaluate this relationship. Consequently, a medium effect size (*d* = 0.50) was used in the power analysis (Cohen, 1988). Additionally, we selected an *α* = 0.05 and an allocation ratio of 2.72 — as the Mann-Whitney *U* test was used to compare 163 heterogeneous sets of WM tasks with 60 homogeneous ones. The results of the analysis indicated that a minimum of 98 heterogeneous and 36 homogenous sets of tasks were required to detect an effect of this size and achieve a statistical power of 0.80. Therefore, our sample was sufficient to compute the planned one-tailed Mann-Whitney *U* test and reach the desired statistical power. A *post hoc* power analysis further validated this, revealing that this analysis achieved a power > 0.99 (*α* = 0.05).

### Design, measure-shortening procedures, and data treatment

The original dataset included 169 participants. Five participants failed to meet the inclusion criteria and were excluded. Another participant scored zero in multiple tasks and was also removed from the sample. As previously mentioned, all data from the multimodal span was excluded from the analyses due to its poor internal consistency (*α* = 0.49).

Thus, we created shortened versions for six WM tasks (reading span, operation span, symmetry span, n-back task, working memory updating task, binding and maintenance task) based on the accuracy scores of 163 participants. These abbreviated versions included the minimum number of trials needed to achieve the conventional threshold for acceptable internal consistency (*α* ≥ 0.70) (Adadan & Savasci, 2012; McDonald, 1999). The length of each shortened WM task was determined through a shortening iterative procedure. In the initial iteration, the reduced versions were based on the first third of trials from the full-length WM tasks^3^. We then calculated the *α* values for these iterations and assessed if they reached the selected cutoff point. *α* values were computed at the level of individual trials with R’s package “psych” (version 2.2.4) (Revelle, 2022). If the first third of trials of a given task did not reach an α of 0.70, we incrementally added two trials to its shortened version until the desired internal consistency was achieved^4^.

After completing the iterative shortening process, the remaining trials were used to form intermediate and later blocks^5^. The number of trials included in these blocks was the same as in the shortened versions/early blocks^6^. For instance, the full-length version of the working memory updating task comprised 12 trials. The first four trials were used to form the early block/shortened version of this task, trials five through eight made up the intermediate block, and the last four trials were used to create the later block. The *α* values and the number of trials included in the complete versions of each WM task and their respective early, intermediate, and later blocks are presented in Table 1.

**Table 1:** Observed and Spearman-Brown prophecy predicted Cronbach’s alphas for every version and each block of the WM tasks

	Full-length version	Early + intermediate blocks	Early block/shortened version	Intermediate block	Later block
RS					
Observed α	0.92 (60)	0.88 (40)	0.78 (20)	0.81 (20)	0.84 (20)
Predicted α	–	0.88	0.79	0.79	0.79
NB					
Observed α	0.82 (12)	–	0.70 (6)	–	0.66 (6)
Predicted α	–	–	0.69	–	0.69
OS					
Observed α	0.90 (60)	0.87 (40)	0.79 (20)	0.80 (20)	0.79 (20)
Predicted α	–	0.86	0.75	0.75	0.75
BT					
Observed α	0.78 (16)	–	0.70 (10)	–	0.72 (10)
Predicted α	–	–	0.69	–	0.69
SS					
Observed α	0.89 (60)	0.85 (40)	0.76 (20)	0.77 (20)	0.76 (20)
Predicted α	–	0.84	0.73	0.73	0.73
WMU					
Observed α	0.93 (12)	0.89 (8)	0.82 (4)	0.82 (4)	0.83 (4)
Predicted α	–	0.90	0.82	0.82	0.82

The number of trials included in each version and block of the WM tasks is presented in parentheses

*RS* reading span, *NB* n-back task, *OS *operation span, *BT* binding and maintenance task, *SS* symmetry span, *WMU* working memory updating task, α Cronbach’s alpha

Then, normalized scores were calculated for the *Gf* tests, the full-length versions of the WM tasks, and their respective early, intermediate, and later blocks. Normalized scores were computed by dividing the number of correct responses by the number of trials included in each task or block (Van Poucke et al., 2016). Given that the shortened WM tasks corresponded to the early trial blocks from the full-length versions of these tests, their scores were equivalent.

Subsequently, we screened the normalized scores for outliers using Microsoft Excel (version 2211). Scores deviating more than 3 SD from the mean were classified as outliers (Ang & Lee, 2010; Lewandowsky et al., 2010). Outliers and zero scores were set to missing. In total, 96 scores (approximately 2% of the data) were reclassified as missing.

Missing values were replaced with plausible scores generated through multiple imputation (Enders & Gottschall, 2011). The guidelines provided by Graham et al. (2007) were used to determine the number of datasets required to replace the missing values. Following these guidelines and considering the highest fraction of missing information in our data (*γ* = 0.10, observed within the scores of the early and later blocks of the n-back task), we generated 20 datasets with multiple imputation and replaced the missing values in our original database.

The imputation process was carried out in RStudio using the “mice” package (version 3.15.0) (Van Buuren & Groothuis-Oudshoorn, 2011). Missing values for each category of variables (early, intermediate, and later blocks of WM tasks, and full-length *Gf* and WM tasks) were imputed using only variables within their respective categories. Furthermore, only significantly correlated variables were included in the regression equations used to calculate the imputed values for each category. For instance, the early blocks of the operation span and the n-back task were not significantly correlated. Therefore, they were not used to impute each other’s missing values. We used the “miceadds” package (version 3.15.21) (Robitzsch & Grund, 2022) to combine the imputed datasets according to Rubin’s (1987) rules. All subsequent analyses were based on the pooled estimates of the imputed databases.

## Statistical analyses and results

Due to the wide range of issues addressed in this study, we have divided this section into several segments. Each segment details the analyses conducted to explore each research question and the respective results. Descriptive statistics for the shortened and full-length versions of the WM tasks and the *Gf* measures are presented in Table 2,^7^. This table also includes the correlations between these tests. The statistics derived from the shortened versions of the WM tasks are displayed in parentheses.

**Table 2:** Descriptive statistics and correlation matrices between the full-length and shortened versions of the WM tests and the Gf tasks

Tasks	Mean	*SD*	Range	Skew	Kurtosis	RS	NB	OS	BT	SS	WMU	LSer	NSer	RAPM
RS	0.76 (.77)	0.16 (0.18)	0.27–1.00 (0.25–1.00)	− 0.97 (− 0.70)	0.45 (− 0.30)	–								
NB	0.62 (.66)	0.23 (0.24)	0.08–1.00 (0.17–1.00)	− 0.23 (− 0.31)	− 0.84 (− 0.85)	0.38^***^ (0.32^***^)	–							
OS	0.76 (0.76)	0.15 (0.18)	0.33–1.00 (0.25–1.00)	− 0.49 (− 0.79)	− 0.34 (0.24)	0.53^***^ (0.35^***^)	0.36^***^ (0.16^*^)	–						
BT	0.81 (0.81)	0.14 (0.16)	0.38–1.00 (0.30–1.00)	− 0.79 (− 0.84)	− 0.04 (0.08)	0.27^***^ (0.28^***^)	0.35^***^ (0.27^***^)	0.26^**^ (0.20^*^)	–					
SS	0.65 (0.65)	0.17 (0.19)	0.23–1.00 (0.15–1.00)	− 0.22 (− 0.28)	− 0.37 (− 0.28)	0.52^***^ (0.39^***^)	0.28^***^ (0.15)	0.42^***^ (0.28^***^)	0.33^***^ (0.21^**^)	–				
WMU	0.56 (0.54)	0.27 (0.29)	0.06–1.00 (0.08–1.00)	0.03 (0.12)	− 1.28 (− 1.29)	0.48^***^ (0.38^***^)	0.38^***^ (0.30^***^)	0.55^***^ (0.42^***^)	0.32^***^ (0.23^**^)	0.54^***^ (0.40^***^)	–			
Lser	0.37	0.14	0.07–0.73	0.15	0.02	0.32^***^ (0.32^***^)	0.28^***^ (0.26^**^)	0.34^***^ (0.24^**^)	0.38^***^ (0.34^***^)	0.29^***^ (0.28^***^)	0.46^***^ (0.50^***^)	–		
Nser	0.41	0.11	0.13–0.80	0.66	0.45	0.37^***^ (0.31^***^)	0.23^**^ (0.26^**^)	0.41^***^ (0.33^***^)	0.32^***^ (0.28^***^)	0.32^***^ (0.31^***^)	0.58^***^ (0.57^***^)	0.47^***^	–	
RAPM	0.48	0.16	0.06–0.89	0.14	− 0.25	0.29^***^ (0.30^***^)	0.30^***^ (0.29^***^)	0.35^***^ (0.24^**^)	0.29^***^ (0.25^**^)	0.41^***^ (0.39^***^)	0.39^***^ (0.33^***^)	0.48^***^	.32^***^	–

*WM* working memory, *Gf* fluid intelligence, *SD* standard deviation, *RS* reading span, *NB* n-back task, *OS* operation span, *BT* binding and maintenance task, *SS* symmetry span, *WMU* working memory updating task,* LSe*r letter series, *NSer* number series, *RAPM* Raven's Advanced Progressive Matrices

^*^p < .05

^**^p < .01

^***^p < .001

### Can WM tasks be reduced and maintain acceptable internal consistency?

We started by examining if the shortened versions of the WM tasks presented acceptable internal consistency. The iterative shortening process reported in the last section confirmed the feasibility of substantially reducing the number of trials per task while maintaining an *α* ≥ 0.70 (Adadan & Savasci, 2012; McDonald, 1999). This procedure suggested that in most cases retaining only the first third of the available trials was sufficient to meet this standard.

Nevertheless, shortening the WM tasks led to a decrease in internal consistency, as evidenced by the values displayed in the third and fifth columns of Table 1. This outcome was expected, given that Cronbach’s *α* is affected by test length (Tavakol & Dennick, 2011). However, to evaluate if the reduction in internal consistency was greater than would be expected, given the number of trials discarded in each task, we computed a Spearman-Brown prophecy (Brown, 1910; Spearman, 1910). This analysis enabled us to determine whether intermediate and later trials contributed more substantially to the internal consistency of the full-length WM tasks than early trials. If that was the case, the actual reduction in internal consistency of the shortened tasks should exceed the prediction of the Spearman-Brown prophecy, given that the intermediate and later blocks of trials were excluded from these versions of the WM tasks. The results of this analysis, presented in Table 1, revealed no significant differences between the *α* predicted by the Spearman-Brown prophecy and the observed *α* of the shortened WM tasks (all absolute *Ws* ≤ 0.45, all *ps* ≥ 0.66, Feldt, 1980). This indicated that the intermediate and later blocks of trials do not contribute more to the internal consistency of WM tasks than earlier blocks. Taken together, these findings suggest that the six WM tasks used in this study can be reduced without compromising their reliability.

### Does shortening the WM tasks significantly impair their ability to predict WMC and Gf?

To assess if reducing the WM tasks significantly decreased their capacity to measure WMC, we derived factor scores from the full-length versions of the six WM tests and evaluated how much variance in these scores was explained by the shortened WM tests. A similar approach was employed to assess the impact of the shortening procedure on their ability to predict *Gf*. In this case, the *Gf* factor scores were derived from three reasoning tests that covered the verbal, numeric, and visuospatial domains typically associated with *Gf* (Unsworth et al., 2009a).

Before addressing these questions, we sought to evaluate if the reasoning tests and the complete versions of the WM tasks measured distinct dimensions. To test this, we computed a SEM to assess whether these tasks loaded on different latent factors (Model 1)^8^. The loadings of all indicators were freely estimated (Beaujean, 2014). The model provided an acceptable fit to the data (Kline, 2015; MacCallum et al., 1996; Marsh et al., 2004), *χ*^*2*^(26) = 55.27, *p* < .001; *χ*^*2*^:*df* = 2.13; CFI = 0.93; RMSEA = 0.08; SRMR = 0.05. All factor loadings were significant (*p* < .001), implying high factor-based reliability. The standardized factor loadings, squared multiple correlations, and standardized error terms are presented in Fig. 2. The *Gf* and WMC factors were highly correlated (*r* = .85, *p* < .001), suggesting that these two sets of tasks measured two distinct, albeit highly related, dimensions. The magnitude of this correlation was similar to that found in previous studies (Hicks et al., 2016; Schmiedek et al., 2009, 2014; Wilhelm et al., 2013).

Subsequently, we derived two sets of orthogonal scores from the *Gf* and WMC factors. The factor scores were calculated using a regression-based approach (Rimm-Kaufman et al., 2015).

To evaluate whether shortening the WM tasks significantly impaired their ability to predict WMC and *Gf*, we computed a series of hierarchical regressions^9^^,^^10^.

Our goal was to examine the impact of the shortening procedure on the entire set of WM tasks and each task individually. To this end, we first tested two hierarchical regression models that estimated the percentage of variance in the WMC and *Gf* factor scores accounted for by the entire set of WM tasks. Following this, we conducted twelve separate hierarchical regressions to assess how well each WM task alone predicted the variance in both sets of factor scores. In each analysis, we initially calculated the amount of *Gf* and WMC variance explained by the early blocks/reduced versions of the WM tasks. We then added the intermediate blocks of each task to the regression models to assess how much additional variance was accounted for by these blocks^11^. At last, we incorporated the later blocks of trials to determine the total variance in the *Gf* and WMC factor scores explained by the early, intermediate, and later blocks of trials. That is, in the final step of the hierarchical regressions, we assessed the extent to which the full-length versions of the WM tasks predicted *Gf* and WMC.

**Fig. 3:**
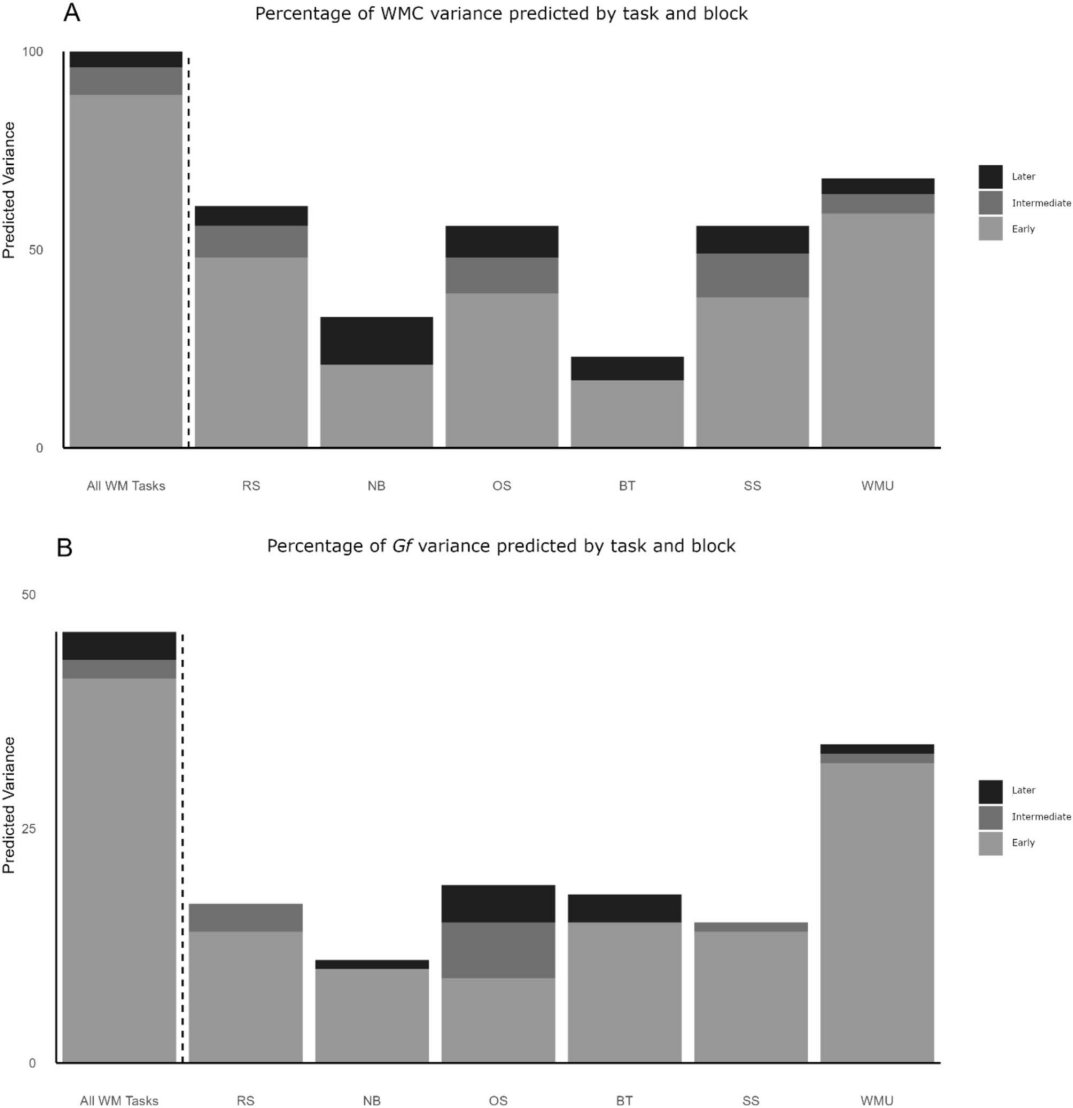
Percentage of variance in the WMC (**A**) and *Gf* factor scores (**B**) predicted by each block of trials from the entire set of WM tasks and each task alone. *RS* reading span, *NB* n-back task, *OS* operation span, *BT* binding and maintenance task, *SS* symmetry span, *WMU* working memory updating task

The regression analyses indicated that all blocks from every task significantly predicted both factor scores (all *ps* < 0.001). The variance explained by the shortened versions/early, intermediate, and later blocks is illustrated in Fig. 3. The bar to the left of the dashed line shows the variance accounted for by the full set of WM tasks. The bars to the right of this line display the variance predicted by each task alone.

As it is possible to observe, the number of trials per block influenced the amount of *Gf* and WMC variance explained, with the full-length task versions accounting for more variance than the shortened versions. However, the regression analyses revealed several interesting patterns. First, the shortened versions (or early blocks) of all WM tasks were able to predict a substantial amount of variance in the WMC factor scores (*R*^*2*^ = 0.90). Additionally, the model that included the full-length versions of all WM tasks (early + intermediate + later blocks of trials) only accounted for 4% of additional *Gf* variance (*R*^*2*^ = 0.46) compared to the model containing all shortened tests (*R*^*2*^ = 0.42). This difference was not significant, *F*(9,147) = 1.28, *p* = .25, suggesting that the shortened and full-length versions of the WM tasks had a similar ability to predict *Gf*. Additionally, using the shortened WM tasks reduces administration time by about 35%, taking only 56 min to complete compared to the 86.7 min required for the full-length versions.

Second, the results of the hierarchical regressions that tested the predictive ability of each WM task individually revealed that, generally, the shortened versions accounted for the majority of the *Gf* and WMC variance explained by the full-length versions of these tasks. This was particularly true regarding their capacity to explain *Gf*. Except for the operation span (*R*^*2*^ shortened version/early block = 0.09, Δ*R*^*2*^ intermediate block = 0.06, Δ*R*^*2*^ later block = 0.04), the intermediate and later blocks either did not predict any additional variance or only accounted for 1% ~ 3% of extra variance in the *Gf* factor scores. The situation was slightly different regarding the tasks’ ability to predict WMC. Although the shortened versions/early blocks accounted for more than half of the variance explained by the complete versions of each task (*R*^*2*^ shortened version/early blocks = 0.17 ~ 59), the intermediate blocks estimated an additional 5–11% of variance, while the later blocks predicted another 4–12%.

The last outcome we want to highlight is that the working memory updating task demonstrated a better predictive ability than any other test. This task accounted for 68% of the variance in the WMC factor scores. This was particularly surprising given that these scores were derived from a factor that included three complex spans and a single version of this task. However, all complex spans accounted for less WMC variance than the working memory updating task (*R*^*2*^ reading span = 0.61, *R*^*2*^ operation span = 0.56, *R*^*2*^ symmetry span = 0.56). The difference in predictive ability between the working memory updating task and the other WM tasks was even more pronounced for *Gf*. The working memory updating task explained nearly twice the variance in the *Gf* factor scores (*R*^*2*^ = 0.34) compared to the binding and maintenance task and the operation span (*R*^*2*^ = 0.19), which were the next best predictors.

In summary, the findings from the regression analyses suggested that multiple tests from the complex span, updating task, and binding task paradigms can be reduced without significantly decreasing their capacity to predict *Gf* and WMC.

### Which combination of block and task should be selected to obtain the best estimate of WMC most efficiently?

The results of the previous analyses revealed that every combination of blocks (early, early + intermediate, early + intermediate + later) from each WM task significantly predicted *Gf* and WMC. This raised the question of which configuration should be selected to maximize the information extracted from the WM tests while minimizing administration time. The answer to this question is not straightforward because the ideal task and block selection varies depending on the aims of the investigation and the time available for administration — some paradigms take longer to complete than others (see Table 3).

**Table 3:** Time (in minutes) needed for subjects to complete each section of the tasks

Minutes to complete each section											
	Calibration Subtask	Instructions	Practice	Early Block	Intermediate Block	Later Block	Short Version	Complete Version			
RS											
Average:	3.78	0.73	0.69	2.78	2.65	2.74	7.98	13.37			
SD:	1.44	1.15	0.31	0.68	0.62	0.73	2.33	3.16			
Median:	3.61	0.54	0.63	2.69	2.60	2.62	7.64	12.93			
Percentile 95:	5.68	1.40	1.16	4.03	3.83	4.19	11.02	19.78			
NB											
Average:	–	2.14	0.50	0.92	–	0.92	3.56	4.48			
SD:	–	3.15	0.22	0.08	–	0.08	3.20	3.20			
Median:	–	1.63	0.45	0.91	–	0.91	3.04	3.79			
Percentile 95:	–	3.20	0.64	0.98	–	0.98	4.83	5.58			
OS											
Average:	3.88	0.67	0.80	2.87	2.78	2.88	8.22	13.88			
SD:	1.57	0.36	0.39	0.72	0.70	1.10	2.46	3.74			
Median:	3.66	0.63	0.70	2.79	2.70	2.75	8.11	13.52			
Percentile 95:	7.07	1.38	1.37	4.20	4.04	4.26	12.82	20.86			
BT											
Average:	–	2.43	0.80	2.32	–	2.30	4.59	6.89			
SD:	–	1.29	0.39	0.10	–	0.09	1.33	1.42			
Median:	–	2.19	0.70	2.30	–	2.29	4.35	6.64			
Percentile 95:	–	4.75	1.34	2.48	–	2.43	7.94	9.27			
SS											
Average:	3.72	1.07	0.78	2.64	2.53	2.60	8.21	13.34			
SD:	1.38	0.51	0.36	0.74	0.64	0.80	2.15	3.24			
Median:	3.57	1.03	0.71	2.46	2.41	2.40	7.78	12.74			
Percentile 95:	5.98	2.03	1.30	3.85	3.60	3.84	12.38	19.51			
WMU											
Average:	–	1.23	1.56	2.19	2.05	2.02	4.97	8.80			
SD:	–	0.64	0.50	0.44	0.35	0.40	1.21	1.60			
Median:	–	1.18	1.43	2.08	1.96	1.94	4.86	8.59			
Percentile 95:	–	2.25	2.54	3.16	2.75	2.59	6.99	11.68			

The column “Calibration Subtask” also includes the time the participants took to read the instructions of this segment

The early block of the binding and maintenance task included the first 10 out of the 16 available trials, while the later block consisted of the last 10 trials. Thus, there was an overlap of four trials between the early and later blocks

All metrics regarding the time it took to complete the early and later blocks of the n-back task are exactly the same because this task was completely computer-paced. That is, both the display time and ISI were fixed in this task. Additionally, the time window allowed for participants to input their responses overlapped with the display time of the stimuli

*RS* reading span, *NB* n-back task, *OS* operation span, *BT* binding and maintenance task, *SS* symmetry span, *WMU* working memory updating task, *SD* standard deviation

To help researchers select the less time-consuming combination best suited to their goals, we performed a permutation analysis in which we calculated the amount of *Gf* and WMC variance predicted by every possible combination of blocks (early, early + intermediate, and early + intermediate + later) and tasks (reading span, operation span, symmetry span, n-back task, working memory updating task, and biding and maintenance task), and the time required to implement each combination.

In total, 2303 models were tested. The results of the permutation analyses are presented in the first three sheets of the Excel file entitled “Original_Results_Permutation_Analysis.xlsx” available on the webpage 10.5281/zenodo.13362470. The sheet “Results Permutation Sorted by Model” contains a table with the results sorted by model complexity. The sheet “Results Permutation Sorted by Time” presents the same information sorted by the time it took to complete the block(s) and task(s) included in each combination. The second column of these tables displays the combination of task(s) and block(s) that were tested in each model. The third column outlines the amount of time it took for 95% of the participants to complete each combination of task(s) and block(s). The fourth and fifth columns display the amount of WMC and *Gf* variance explained by each combination. Finally, the sixth column contains the proportion of *Gf* variance predicted by the full set of complete WM tasks (45.7%) that each combination of block(s) and task(s) accounted for. We calculated this metric to get a clearer understanding of the impact of the reduction tested in each model. In the sheet “Results Permutation Sorted by Time” of the Excel file, the models that account for the most *Gf* and WMC variance in the least amount of time are highlighted in light grey.

To provide a general answer to the question of which blocks and tasks should be used to gain the most information from the WM tasks in the least amount of time, we adopted and expanded the criteria used by Foster et al. (2015). These authors considered that a combination of blocks and tasks provided a satisfactory estimate of WMC if it was able to account for at least 90% of the *Gf* variance explained by the full model, which in their case included the full-length versions of three complex spans. In our study, we expanded this criterion and only considered that a model was a valid measure of WMC if it was able to explain 90% of the variance in both WMC and *Gf* factor scores. The sheet “Models Explaining > 90% Variance” of the Excel file contains the models that met this criterion. The results of the permutation analysis indicated that the model that included the complete versions of the reading span, binding and maintenance task, and working memory updating task was the one that met this requirement while minimizing administration time. This combination took less than half the time to complete (40.7 min) compared to the entire set of full-length WM tasks (86.7 min) and was still able to predict 90% of the WMC variance and 93% of the *Gf* variance accounted for by the full model.

However, this combination may be construed as the most cost-effective solution if we only consider this criterion. As mentioned earlier, optimal task and block selection depends on various factors, including the time available for test administration and the specific objectives of the investigation. To help researchers select the combinations best suited to their investigative needs, we developed an interactive web application, accessible at https://shortwmtasks.shinyapps.io/Results_Permutation_Analysis/. This tool allows users to customize their selection criteria based on the specific constraints of their research. Researchers can filter models by factors such as available testing time, the desired amount of *Gf* and WMC variance explained, the targeted WM components, and the inclusion or exclusion of specific tasks.

Therefore, the information provided in the web application and in the first three sheets of the file “Original_Results_Permutation_Analysis.xlsx” may help researchers determine the best combination of tasks and blocks given the specific requirements of their study. For instance, if the researcher only has 20 min to implement the WM tasks, the permutation analysis suggested that the most effective solution would be to use the early block of the binding and maintenance task and the complete version of the working memory updating task. However, if the researcher can allot 40 min to this process, the ideal combination would be to employ the early block of the reading span and the full-length versions of the n-back task, the binding and maintenance task, and the working memory updating task.

Besides offering general guidelines that may help researchers select the most appropriate combination of tasks and blocks, the results of the permutation analysis provided some additional insights regarding the optimal administration of WM measures. For instance, several sources state that the complex spans are the most widely used tests to assess WMC (Conway et al., 2005; Gonthier et al., 2016; Wilhelm et al., 2013). In fact, numerous studies have employed either a single (Al-Rashidi et al., 2023; Unsworth & Engle, 2005) or multiple variants (Burgoyne et al., 2023; Engle et al., 1999; Kane et al., 2004) of this paradigm to measure this construct. However, this class of tasks may not be the most effective to get the best estimate of WMC.

According to our analysis, each complex span took around 20 min to complete and accounted for 55.8 ~ 60.6% of the variance in the WMC factor scores and 33.7% ~ 40.9% of the *Gf* variance explained by the full model. In contrast, while taking virtually the same amount of time to complete, the combination of the early block of the binding and maintenance task and the complete version of the working memory updating task was able to predict 79.8% of the variance in the WMC factor scores and 78.4% of the *Gf* variance accounted for by the full set of complete WM tasks. Additionally, this combination derives WMC from two indicators which facilitate the separation of construct-relevant variance from confounding sources of variation (Gonthier et al., 2016; Waris et al., 2017). Furthermore, completing the three complex spans took around 60 min and estimated 88.9% of the WMC variance and 63.6% of the *Gf* variance predicted by the complete version of all WM tasks. However, our analysis revealed that if the researchers can allot 60 min to the administration of WM tasks, using the blocks and tasks included in Model 2013 (early blocks of the n-back and binding and maintenance tasks, early + intermediate blocks of the operation span, and the full-length versions of the symmetry span and the working memory updating task) will produce a better estimate of WMC. The blocks included in this model explained 92.7% of the WMC variance and 96.4% of the *Gf* variance explained by the full model.

### Do heterogeneous and homogenous sets of tasks differ significantly in their capacity to predict Gf?

The outcome of the permutation analyses revealed that the multidimensional set of tasks tested in model 2013 had a better predictive ability than the combination of the complete versions of the three complex spans included in the OpenWMB. This finding aligns with previous research, which suggested that implementing multiple indicators that differ in construct-irrelevant features provides a better indirect representation of a cognitive concept than applying a set of similar tasks that share most of the construct-relevant characteristics (Little et al., 1999; Schmiedek et al., 2014). Considering this, we evaluated if this outcome could be generalized. That is, we assessed whether heterogeneous models composed of tasks from different paradigms were better predictorsof *Gf* than homogenous models that only included measures from the same class of WM tasks^12^.

To test this hypothesis, we selected the 163 combinations of triplets and duos that only included tasks from different paradigms (complex spans, updating tasks, and binding tasks) and the 60 models comprising triplets and duos of measures from the same category. In the case of the heterogeneous models, each triplet contained one task from each paradigm, and each duo contained tasks from two different paradigms. These models are listed in the sheet “Models Mann Whitney” of the Excel file entitled “Original_Results_Permutation_Analysis.xlsx”. We then computed a Mann-Whitney *U* test^13^ to evaluate if the heterogeneous and homogenous sets of tasks differed in their ability to predict *Gf*. The analysis revealed a statistically significant difference between the average percentage of *Gf* variance predicted by the heterogeneous models (mean *R*^*2*^ = 0.33) and the homogeneous sets of tasks (mean *R*^*2*^ = 0.25), *U* = 7712.50, *p* < .001, *r* = .44. Thus, these findings indicated that, in general, applying a heterogeneous set of WM tasks incorporating measures from different paradigms generates a better estimate of WMC than only using tests from the same class.

## Discussion

The primary goal of this study was to determine whether WM tasks from the complex span (reading, operation, and symmetry spans), updating task (n-back and working memory updating tasks), and binding task (binding and maintenance task) paradigms could be effectively shortened and preserve robust psychometric proprieties. Our analysis demonstrated that the observed *α* values of the shortened WM tasks met or surpassed the conventional threshold for acceptable internal consistency (all *αs* ≥ 0.70) (Adadan & Savasci, 2012; McDonald, 1999). These values were comparable to, and in some cases exceeded, those reported in previous studies (Felez-Nobrega et al., 2018; Foster et al., 2015; Gonthier et al., 2016; Oswald et al., 2015). Additionally, the shortened tasks accounted for the vast majority of the variance in a set of WMC factor scores derived from the full-length versions of the WM tests (*R*^*2*^ = 0.90). Furthermore, there was no significant difference between the shortened and full-length versions in their ability to predict *Gf*. These findings were consistent with the results reported by Foster et al. (2015). The shortened WM tasks reduced the total administration time by approximately 35%, requiring only 56 min to complete compared to 86.7 min for the full-length versions.

A notable finding from this study is that updating and binding tasks can be effectively reduced without compromising their psychometric integrity. To the best of our knowledge, this is the first study that evaluated this possibility. Previous studies with similar objectives primarily focused on complex spans (Felez-Nobrega et al., 2018; Foster et al., 2015; Gonthier et al., 2016; Oswald et al., 2015). Complex spans are highly reliable and valid tests, with numerous investigations employing either a single (Al-Rashidi et al., 2023; Unsworth & Engle, 2005) or multiple variants (Burgoyne et al., 2023; Engle et al., 1999; Kane et al., 2004) of this paradigm to measure WMC. However, estimating WMC based on a single task or paradigm risks conflating variation due to individual differences in WMC with task- and paradigm-specific variance (Lewandowsky et al., 2010; Schmiedek et al., 2014). Administering multiple tasks from different paradigms helps to separate these confounding influences from pure WMC variance, resulting in a more accurate measurement of this construct (Waris et al., 2017; Wilhelm et al., 2013). However, this approach can be time-consuming, as the participants must read different sets of instructions and adapt to various task structures. By creating shortened versions of WM tasks from different paradigms, we provide researchers with a method to obtain robust WMC estimates free of task- and paradigm-specific variance, at a reduced time cost.

It is important to note that using all shortened WM tasks is not necessary for an accurate WMC measurement. Depending on the specific aims and time available for task administration, different combinations of shortened and/or full-length WM tasks can be used to obtain reliable WMC estimates in a time-efficient manner. To assist researchers in selecting the configuration most suited to their needs, we computed a permutation analysis in which we assessed the efficacy of every possible combination of blocks and tasks. This analysis builds on the work of Foster et al. (2015), who examined how well different combinations of blocks of trials (early, early + intermediate, and early + intermediate + later) derived from three complex span tasks predicted a set of *Gf* factor scores and calculated the time required to complete each combination. However, they only tested tasks from a single paradigm and administered these tests in a fixed order, limiting the number of combinations they could assess (39). We included six tasks from three different paradigms in this analysis. Additionally, we did not have to account for possible order effects as we counterbalanced the order of presentation of the tasks in our study. This allowed us to test 2303 models.

The results of the permutation analysis suggested that employing the complete versions of the reading span, binding and maintenance task, and working memory updating task provided the best WMC estimate in the least amount of time based on the criterion that we adopted — only combinations that were able to explain 90% of the variance in both WMC and *Gf* factor scores were considered acceptable measures of WMC. This set of tasks took less than half the time to complete (40.7 min) compared to the entire set of full-length WM tasks (86.7 min) and was able to predict 90% of the WMC variance and 93% of the *Gf* variance accounted for by the full model. Thus, our findings suggested that researchers who intend to extract the most amount of WMC variance in the least amount of time should apply this set of tasks.

However, we would like to point out that this conclusion is based on the criterion adopted in this study. As previously stated, optimal task selection should be guided by the specific objectives and constraints of each investigation. Considering this, we provided two resources (the Excel file ‘Original_Results_Permutation_Analysis.xlsx,’ located at 10.5281/zenodo.13362470 and the interactive web application hosted in https://shortwmtasks.shinyapps.io/Results_Permutation_Analysis/) that provide detailed information to help researchers identify the most suitable combination of tasks for their study. Researchers can use these resources to tailor their selection based on available testing time, the desired amount of *Gf* and WMC variance explained, the targeted WM components, and the inclusion or exclusion of specific tasks.

We have previously outlined examples detailing how task selection can be optimized based on the time available for task administration. For instance, if only 20 min are available, the permutation analysis indicates that the most effective approach would involve implementing the initial block of the binding and maintenance task alongside the complete version of the working memory updating task. Conversely, if the researcher has 40 min to administrate the WM tasks, the analysis suggests that the optimal combination would include the initial block of the reading span task, as well as the full-length versions of the n-back task, the binding and maintenance task, and the working memory updating task.

If the goal of the investigation is to obtain a multi-indicator estimate of the updating component of WM, the results of the permutation analysis indicated that administering the shortened versions of the n-back task and the working memory updating task is enough to account for 97% of the WMC and *Gf* variance explained by their full-length counterparts while reducing testing time by approximately 5 min. Alternatively, if the objective is to assess the ability to simultaneously store and process information, the researcher can account for 90% of the WMC and *Gf* variance explained by the full-length versions of the three complex span tasks by implementing only the early and intermediate blocks of the symmetry span and the full-length operation span. This approach saves approximately 24 min.

Thus, the results of the permutation analysis provide valuable insights for researchers, highlighting ways to achieve robust WMC estimates while minimizing testing time. Furthermore, they offer more efficient alternatives to the prevailing approach of estimating WMC with single or multiple versions of the complex span (Burgoyne et al., 2023; Engle et al., 1999; Al-Rashidi et al., 2023; Kane et al., 2004; Unsworth & Engle, 2005).

In addition to offering new methods to improve the time efficiency of WMC assessment, this study also contributes to other questions related to the optimal administration of WM tasks. For example, we explored whether heterogeneous combinations of tests from different paradigms and homogenous sets of tasks from the same class differed in their ability to predict *Gf* — a commonly used method to assess the criterion validity of WM tasks (Lewandowsky et al., 2010; Schmiedek et al., 2009). Our analysis revealed that heterogeneous combinations provided better representations of WMC than homogenous ones. These results align with the findings of Schmiedek et al. (2014). The superior predictive ability of heterogeneous combinations may be explained by the inclusion of indicators that covered wider sources of construct-relevant variation (Little et al., 1999): complex spans involve simultaneous storage and processing activities (Redick et al., 2012; Unsworth et al., 2009a, b), updating tasks require ongoing renewal of mental representations (Ecker et al., 2010), and binding tasks involve associating multiple stimulus features to create new relationships (Oberauer et al., 2003). Combinations that only included a single paradigm probably overlooked some of these dimensions. Moreover, as previously stated, using heterogeneous combinations helps to distinguish construct-relevant from task- and paradigm-specific variance, allowing for a more precise interpretation of WMC measurements (Gonthier et al., 2016; Waris et al., 2017).

One particularly surprising finding was that the working memory updating task predicted both WMC and *Gf* factor scores more accurately than any other measure. This was unexpected, given that the WMC factor scores were derived from a set of tests including three complex spans and a single version of the working memory updating task. This suggested that the updating processes assessed by this task may be particularly important for estimating both WMC and *Gf* (Ecker et al., 2010). Traditionally, the complex span has been the most widely used paradigm to measure WMC (Conway et al., 2005; Redick et al., 2012). However, our analyses indicate that the working memory updating task may provide more accurate estimates of WMC than any complex span.

While our research offers new perspectives for WMC assessment, it is important to acknowledge some limitations that may have influenced our findings. Future research might address these gaps to refine and build upon our results.

For instance, we only administered the WM tasks once in their full-length form. These tests were then segmented into blocks representing early, intermediate, and later trials. The shortened versions of the WM tasks were based on the blocks containing the early trials of each test. Thus, we did not administer the shortened versions by themselves. It would be important to assess whether the shortened WM tasks present similar levels of reliability and validity as those reported in this article when administered in isolation. One way to examine this would be to implement a split-sample design. In this approach, the sample would be divided into two groups: one group would complete both the full-length and shortened versions of the WM tasks, while the other would only complete the shortened versions. The first group would allow for direct comparisons of the psychometric properties between the two formats within the same individuals. On the other hand, the second group would provide insights into the reliability and validity of the shortened tasks in conditions more closely resembling their intended use in future studies. Moreover, it would be interesting to extend this approach to diverse populations, such as children, the elderly, and clinical groups, to determine if the shortened WM tasks provide reliable and valid WMC estimates across different populations.

The single administration of the shortened WM tasks introduces another potential limitation: while this version effectively explained the vast majority of the variance in the full-length tasks, supporting their utility in cross-sectional studies that seek to assess WMC, their ability to capture significant within-person variability over time remains uncertain. If the shortened tasks can reliably capture such variations, they could be particularly valuable for longitudinal research in cognitive psychology (e.g., behavioral studies investigating diurnal fluctuations in WM performance (Schmidt et al., 2015; Van Eekelen & Kerkhof, 2003) and neuroscience (e.g., neuroimaging investigations seeking to build personalized brain and behavior models to identify psychiatric biomarkers (Kraus et al., 2023; Makowski et al., 2024)). The shortened WM tasks could serve as a time-efficient alternative for repeated assessments of WM performance, facilitating the detection of intra-individual changes over time while maintaining the quality of the measurements and minimizing participant fatigue. To address this limitation, future research should explore whether the short WM tasks are able to effectively capture within-person variability in WM performance if they are administered multiple times within the same day and across multiple days.

Another aspect that needs to be addressed in future studies is the uneven distribution of paradigms in this study: our participants completed three complex spans, two updating tasks, and a single binding task. As a result, the factor scores derived from these tasks were probably not the most balanced representation of WMC. Considering the features of these paradigms, it is possible that these scores were overly influenced by the ability to simultaneously process and store information while underrepresenting the capacity to bind different stimulus features. This limitation may explain why the binding and maintenance task had the lowest ability to predict the WMC factor scores.

We also chose not to use the WMC factor scores as a criterion when comparing the predictive ability of heterogeneous and homogeneous task sets because of this uneven representation of WM paradigms. We made this decision because 54 out of the 60 homogeneous models we tested were composed of complex spans, which could have biased the results. Future studies with similar goals should consider administering a balanced set of WM tests, with an equal number of tasks per paradigm, as this will allow the use of both WM and *Gf* factor scores to determine whether heterogeneous or homogeneous test sets provide a better method for measuring WMC.

Another drawback of applying only a single binding task was the inability to confirm whether the robust metrics of the reduced version of the binding and maintenance task could be generalized to other tests within this paradigm. Future studies should explore this issue further.

Beyond the tasks themselves, another aspect that needs to be considered is the optimization of task instructions. The time required to read these instructions often consumes a significant portion of the administration time (Foster et al., 2015). In our study, 95% of participants took approximately 15 min to read the instructions, constituting 17% of the total administration time for the full-length tests and 27% for the shortened versions. Future research should examine whether streamlining the instructions can reduce reading time without sacrificing clarity.

## Conclusion

Traditionally, assessing WMC relies on extensive cognitive tests comprising numerous trials (Stollery & Christian, 2016; Unsworth et al., 2005; Waris et al., 2021). The reason for including such a high number of trials is that most WM tasks were initially developed as independent measures capable of providing reliable and valid estimates of WMC on their own (Gonthier et al., 2016). However, our findings suggested that it is possible to substantially shorten tests from multiple paradigms and preserve their good psychometric proprieties. Implementing the shortened versions resulted in a 35% reduction in administration time, equivalent to a time savings of approximately 30 min. By creating reduced versions of WM tasks from different paradigms, we offer researchers an efficient solution to obtain accurate WMC estimates free of task- and paradigm-specific variance, at a reduced time cost. Additionally, the results of the permutation analysis identified less time-consuming combinations of shortened and/or full-length tasks that yield more robust WMC estimates than the conventional approach of using either a single or multiple versions of the complex span. We believe that these shortened WM tasks will be highly valuable for researchers, enabling more time to assess additional constructs and enhancing participant recruitment through quicker data collection.

## Data Availability

All code, data, and materials mentioned in this article are available on the webpages https://doi.org/10.5281/zenodo.13362470 and https://shortwmtasks.shinyapps.io/Results_Permutation_Analysis/. The first webpage contains a folder entitled “Shortened_OpenWMB.zip,” which includes a script with the shortened versions of the WM tasks. If the URL of the interactive application changes, please visit our Zenodo page for the updated adress.

## Footnotes

1 The OpenWMB includes an automated version of the multimodal span (Quinette et al., 2006). However, the full-length version of this task presented low internal consistency (α = 0.49). Considering that one of the aims of this study was to shorten the WM tasks while maintaining an *α* ≥ 0.70, and that not even the full version of the multimodal span was able to meet this requirement, we excluded all data from this task from further analyses.

2 In this article, we use the term “trial” to describe a single instance in which a stimulus is presented and processed according to the requirements of the task (e.g., in the reading span, a trial comprises the presentation of a sentence, assessing if the sentence contains a syntactical error, and the presentation and memorization of the to-be-remembered letter). We use the term “block” to define a set of trials.

3 In the initial iteration of the shortening procedure, the early blocks were formed by the first third of trials of each task due to the structure of the complex spans. Each complex span comprised three blocks of trials with sizes ranging from two to six. To preserve this range of block sizes in their shortened versions, we needed to include at least the first block of trials of each length.

4 Two trials were added in each iteration because most tasks presented a balanced design (e.g., in the symmetry span, half of the matrices were symmetrical, and the other half were asymmetrical). Adding a single trial in each iteration would disrupt this balance.

5 The complete versions of the n-back and the binding and maintenance task contained 12 and 16 scored trials, respectively. Given the number of trials needed to create their early blocks and achieve an *α* ≥ 0.70 (6 and 10, respectively), we were unable to generate intermediate blocks of trials for these tasks. Additionally, the early and later blocks of the binding and maintenance task shared 4 trials. Therefore, the results of analyses involving these blocks should be interpreted with caution.

6 Throughout this text we will use the terms “shortened version(s)” and “early block(s) of trials” interchangeably.

7 The descriptive statistics and correlational analyses were computed in R with the packages “stats” (version 4.4.1) (R Core Team, 2013), “Hmisc” (version 5.1.3) (Harrell Jr & Dupont, 2024), and “MVN” (version 5.9) (Korkmaz et al., 2022).

8 This analysis was computed in R with the packages “lavaan” (version 0.6.18) (Rosseel et al., 2012), “semPlot” (version 1.1.6) (Epskamp, 2022), and “MVN” (version 5.9) (Korkmaz et al., 2022). All factors were extracted using maximum likelihood and subjected to an oblique rotation (promax) due to the continuous nature and the univariate and multivariate normal distribution of our data (Fabrigar et al., 1999; Kline, 2015). The distribution of all observed variables presented low values of skewness (< 2) and kurtosis (< 4) (Kline, 2015). Additionally, Mardia’s coefficients (all *β*_*2*_*s* < 3, all *ps* > 0.05) indicated an absence of multivariate kurtosis (Romeu & Ozturk, 1993).

9 Regression analyses were implemented in R, with the package “stats” (version 4.4.1) (R Core Team, 2013) and “nmle” (version 3.1.165) (Pinheiro et al., 2024).

10 Models were estimated using weighted least squares when heteroscedasticity was present in the data (Field, 2017). Generalized least squares were used when there was evidence of autocorrelation (Beguería & Pueyo, 2009). All other models were computed using ordinary least squares.

11 The shortening iterative process did not allow us to create intermediate blocks for the n-back and the binding and maintenance tasks. Consequently, no intermediate blocks from these tasks were added in step 2 of the hierarchical regression analyses. Additionally, the shortened version/early block of the binding and maintenance task included 10 trials, while the complete version comprised 16. Thus, only six additional trials were included in the regression models that incorporated the full-length version of this task.

12 We chose not to test if the heterogenous and homogenous sets of tasks differed in their ability to predict WMC because the factor scores representing this construct were derived from a set of six tasks that included three complex spans. Moreover, 54 out of the 60 homogenous models that we tested were composed of complex spans. Thus, we believe that the results of this analysis could have led to a biased interpretation.

13 This analysis was performed in R with the package “rstatix” (version 0.7.2) (Kassambara, 2023). We used a Mann-Whitney *U* test because there was evidence of heteroscedasticity between the heterogenous and homogenous sets of tasks, *F(*1,221) = 35.35, *p* < .001.

## References

[CR1] Adadan, E., & Savasci, F. (2012). An analysis of 16-17-year-old students’ understanding of solution chemistry concepts using a two-tier diagnostic instrument. *International Journal of Science Education*, *34*(4), 513–544. 10.1080/09500693.2011.636084

[CR2] Al-Rashidi, A., Vadivel, B., Khalil, N., & Basim, N. (2023). The comparative impacts of portfolio-based assessment, self-assessment, and scaffolded peer assessment on reading comprehension, vocabulary learning, and grammatical accuracy: Insights from working memory capacity. *Language Testing in Asia,**13*(1), 24. 10.1186/s40468-023-00237-1

[CR3] Allen, K., Giofrè, D., Higgins, S., & Adams, J. (2020). Working memory predictors of mathematics across the middle primary school years. *British Journal of Educational Psychology*, *90*(3), 848–869. 10.1111/bjep.12339 31999851 10.1111/bjep.12339 PMC7496726

[CR4] Ang, S. Y., & Lee, K. (2010). Exploring developmental differences in visual short-term memory and working memory. *Developmental Psychology*, *46*(1), 279–285. 10.1037/a0017554 20053024 10.1037/a0017554

[CR5] Baddeley, A. (2012). Working memory: Theories, models, and controversies. *Annual Review of Psychology*, *63*, 1–29. 10.1146/annurev-psych-120710-100422 21961947 10.1146/annurev-psych-120710-100422

[CR6] Barkus, E. (2020). Effects of working memory training on emotion regulation: Transdiagnostic review. *PsyCh Journal*, *9*(2), 258–279. 10.1002/pchj.353 32166891 10.1002/pchj.353

[CR7] Bartsch, L., Loaiza, V., & Oberauer, K. (2018). Does limited working-memory capacity underlie age differences in associative long-term memory? *Psychology and Aging*, *34*(2), 268–281. 10.1037/pag0000317 30407033 10.1037/pag0000317

[CR8] Beaujean, A. A. (2014). *Latent variable modeling using R* (1st ed.). Routledge.

[CR9] Beguería, S., & Pueyo, Y. (2009). A comparison of simultaneous autoregressive and generalized least squares models for dealing with spatial autocorrelation. *Global Ecology and Biogeography*, *18*(3), 273–279. 10.1111/j.1466-8238.2009.00446.x

[CR10] Brown, W. (1910). Some experimental results in the correlation of mental abilities. *British Journal of Psychology*, *3*, 296–322. 10.1111/j.2044-8295.1910.tb00207.x

[CR11] Burgoyne, A. P., Mashburn, C. A., Tsukahara, J. S., Hambrick, D. Z., & Engle, R. W. (2023). Understanding the relationship between rationality and intelligence: A latent-variable approach. *Thinking and Reasoning*, *29*(1), 1–42. 10.1080/13546783.2021.2008003

[CR12] Cohen, J. (1988). *Statistical power analysis for the behavioural sciences* (2nd ed.). Academic.

[CR13] Colom, R., Martínez-Molina, A., Shih, P. C., & Santacreu, J. (2010). Intelligence, working memory, and multitasking performance. *Intelligence*, *38*(6), 543–551. 10.1016/j.intell.2010.08.002

[CR14] Conway, A. R., Kane, M. J., Bunting, M. F., Hambrick, D. Z., Wilhelm, O., & Engle, R. W. (2005). Working memory span tasks: A methodological review and user’s guide. *Psychonomic Bulletin & Review*, *12*(5), 769–786. 10.3758/BF03196772 16523997 10.3758/bf03196772

[CR15] Daneman, M., & Carpenter, P. A. (1980). Individual differences in working memory and reading. *Journal of Verbal Learning and Verbal Behavior*, *19*(4), 450–466. 10.1016/S0022-5371(80)90312-6

[CR16] Ecker, U. K., Lewandowsky, S., Oberauer, K., & Chee, A. E. (2010). The components of working memory updating: An experimental decomposition and individual differences. *Journal of Experimental Psychology: Learning Memory and Cognition*, *36*(1), 170–189. 10.1037/a0017891 20053053 10.1037/a0017891

[CR17] Enders, C. K., & Gottschall, A. C. (2011). Multiple imputation strategies for multiple group structural equation models. *Structural Equation Modeling*, *18*(1), 35–54. 10.1080/10705511.2011.532695

[CR18] Engle, R. W., Laughlin, J. E., Tuholski, S. W., & Conway, A. R. (1999). Working memory, short-term memory, and general fluid intelligence: A latent-variable approach. *Journal of Experimental Psychology: General*, *128*(3), 309–331. 10.1037/0096-3445.128.3.309 10513398 10.1037//0096-3445.128.3.309

[CR19] Epskamp, S. (2022). semPlot: path diagrams and visual analysis of various SEM packages output. R package version 1.1.6. Retrieved from: https://cran.r-project.org/web/packages/semPlot/index.html

[CR20] Fabrigar, L. R., Wegener, D. T., Maccallum, R. C., & Strahan, E. J. (1999). Evaluating the use of exploratory factor analysis in psychological research. *Psychological Methods*, *4*(3), 272–299. 10.1037/1082-989X.4.3.272

[CR21] Faul, F., Erdfelder, E., Buchner, A., & Lang, A. G. (2009). Statistical power analyses using G*Power 3.1: Tests for correlation and regression analyses. *Behavior Research Methods*, *41*(4), 1149–1160. 10.3758/BRM.41.4.1149 19897823 10.3758/BRM.41.4.1149

[CR22] Feldt, L. S. (1980). A test of the hypothesis that Cronbach’s alpha reliability coefficient is the same for two tests administered to the same sample. *Psychometrika*, *45*(1), 99–105. 10.1007/BF02293600

[CR23] Felez-Nobrega, M., Foster, J. L., Puig-Ribera, A., Draheim, C., & Hillman, C. H. (2018). Measuring working memory in the Spanish population: Validation of a multiple shortened complex span task. *Psychological Assessment*, *30*(2), 274–279. https://doi.org/10.1037/ pas0000484 28406672 10.1037/pas0000484

[CR24] Field, A. (2017). *Discovering statistics using IBM SPSS* (5th ed.). SAGE.

[CR25] Foster, J. L., Shipstead, Z., Harrison, T. L., Hicks, K. L., Redick, T. S., & Engle, R. W. (2015). Shortened complex span tasks can reliably measure working memory capacity. *Memory and Cognition*, *43*(2), 226–236. 10.3758/s13421-014-0461-7 25217113 10.3758/s13421-014-0461-7

[CR26] Friedman, N. P., & Miyake, A. (2004). The reading span test and its predictive power for reading comprehension ability. *Journal of Memory and Language*, *51*(1), 136–158. 10.1016/j.jml.2004.03.008

[CR27] Garcia, R. B., Mammarella, I. C., Tripodi, D., & Cornoldi, C. (2014). Visuospatial working memory for locations, colours, and binding in typically developing children and in children with dyslexia and non-verbal learning disability. *British Journal of Developmental Psychology*, *32*(1), 17–33. 10.1111/bjdp.12019 25284471 10.1111/bjdp.12019

[CR28] Gonthier, C., Thomassin, N., & Roulin, J. L. (2016). The composite complex span: French validation of a short working memory task. *Behavior Research Methods*, *48*(1), 233–242. 10.3758/s13428-015-0566-3 25669761 10.3758/s13428-015-0566-3

[CR29] Graham, J. W., Olchowski, A. E., & Gilreath, T. D. (2007). How many imputations are really needed? Some practical clarifications of multiple imputation theory. *Prevention Science*, *8*(3), 206–213. 10.1007/s11121-007-0070-9 17549635 10.1007/s11121-007-0070-9

[CR30] Grant, D. A. (1948). The latin square principle in the design and analysis of psychological experiments. *Psychological Bulletin*, *45*(5), 427. 10.1037/h0053912 18885731 10.1037/h0053912

[CR31] Gray, S., Green, S., Alt, M., Hogan, T., Kuo, T., Brinkley, S., & Cowan, N. (2017). The structure of working memory in young children and its relation to intelligence. *Journal of Memory and Language*, *92*, 183–201. 10.1016/j.jml.2016.06.004 27990060 10.1016/j.jml.2016.06.004 PMC5157932

[CR32] HarrellJr, F. E., & Dupont, C. (2024). Hmisc: Harrell miscellaneous. R package version 5.1.3. Retrieved from: https://cran.r-project.org/web/packages/Hmisc/index.html

[CR33] Heitz, R. P., Schrock, J. C., Payne, T. W., & Engle, R. W. (2008). Effects of incentive on working memory capacity: Behavioral and pupillometric data. *Psychophysiology*, *45*(1), 119–129. 10.1111/j.1469-8986.2007.00605.x 17910734 10.1111/j.1469-8986.2007.00605.x

[CR34] Hicks, K. L., Foster, J. L., & Engle, R. (2016). Measuring working memory capacity on the web with the Online Working Memory Lab (the OWL). *Journal of Applied Research in Memory and Cognition*, *5*, 478–489. 10.1016/j.jarmac.2016.07.010

[CR35] Kane, M. J., Tuholski, S. W., Hambrick, D. Z., Wilhelm, O., Payne, T. W., & Engle, R. W. (2004). The generality of working memory capacity: A latent-variable approach to verbal and visuospatial memory span and reasoning. *Journal of Experimental Psychology: General*, *133*(2), 189–217. 10.1037/0096-3445.133.2.189 15149250 10.1037/0096-3445.133.2.189

[CR36] Kassambara, A. (2023). rstatix: pipe-friendly framework for basic statistical tests. R package version 0.7.2. Retrieved from: https://cran.r-project.org/web/packages/rstatix/index.html

[CR37] Kattner, F. (2021). Transfer of working memory training to the inhibitory control of auditory distraction. *Psychological Research,**85*(8), 3152–3166. 10.1007/s00426-020-01468-0 33449207 10.1007/s00426-020-01468-0 PMC8476394

[CR38] Kieslich, P. J., & Henninger, F. (2017). Mousetrap: An integrated, open-source mouse-tracking package. *Behavior Research Methods*, *49*(5), 1652–1667. 10.3758/s13428-017-0900-z 28646399 10.3758/s13428-017-0900-z

[CR39] Kirchner, W. K. (1958). Age differences in short-term retention of rapidly changing information. *Journal of Experimental Psychology*, *55*(4), 352–358. 10.1037/h0043688 13539317 10.1037/h0043688

[CR40] Kline, R. B. (2015). *Principles and practice of structural equation modeling* (5th ed.). The Guilford Press.

[CR41] Korkmaz, S., Goksuluk, D., & Zararsi, G. (2022). MVN: multivariate normality tests. R package version 5.9. Retrieved from: https://cran.r-project.org/web/packages/MVN/

[CR42] Kraus, B., Zinbarg, R., Braga, R. M., Nusslock, R., Mittal, V. A., & Gratton, C. (2023). Insights from personalized models of brain and behavior for identifying biomarkers in psychiatry. *Neuroscience & Biobehavioral Reviews*, *152*, 105259. 10.1016/j.neubiorev.2023.105259 37268180 10.1016/j.neubiorev.2023.105259 PMC10527506

[CR43] Lewandowsky, S., Oberauer, K., Yang, L. X., & Ecker, U. K. (2010). A working memory test battery for MATLAB. *Behavior Research Methods*, *42*(2), 571–585. 10.3758/BRM.42.2.571 20479189 10.3758/BRM.42.2.571

[CR44] Li, X., Yi, Z., Lv, Q., Chu, M., Hu, H., Wang, J., Zhang, J., Cheung, E. E., & Chan, R. C. (2019). Clinical utility of the dual n-back task in schizophrenia: A functional imaging approach. *Psychiatry Research - Neuroimaging*, *284*, 37–44. 10.1016/j.pscychresns.2019.01.002 30658243 10.1016/j.pscychresns.2019.01.002

[CR45] Little, T. D., Lindenberger, U., & John, R. (1999). On selecting indicators for multivariate measurement and modeling with latent variables: When good indicators are bad and bad indicators are good. *Psychological Methods*, *4*(2), 192–211. 10.1037/1082-989X.4.2.192

[CR46] Ma, L., Chang, L., Chen, X., & Zhou, R. (2017). Working memory test battery for young adults: Computerized working memory assessment. *Plos One,**12*(3), e0175047. 10.1371/journal.pone.0175047 28362867 10.1371/journal.pone.0175047 PMC5376327

[CR47] MacCallum, R. C., Browne, M. W., & Sugawara, H. M. (1996). Power analysis and determination of sample size for covariance structure modeling. *Psychological Methods*, *1*(2), 130–149. 10.1037/1082-989X.1.2.130

[CR48] Makowski, C., Nichols, T. E., & Dale, A. M. (2024). Quality over quantity: Powering neuroimaging samples in psychiatry. *Neuropsychopharmacology : Official Publication of the American College of Neuropsychopharmacology*, *50*, 58–66. 10.1038/s41386-024-01893-4 38902353 10.1038/s41386-024-01893-4 PMC11525971

[CR49] Marsh, H. W., Hau, K. T., & Wen, Z. (2004). In search of golden rules: Comment on hypothesis-testing approaches to setting cutoff values for fit indexes and dangers in overgeneralizing Hu and Bentler’s (1999) findings. *Structural Equation Modeling*, *11*(3), 320–341. 10.1207/s15328007sem1103_2

[CR50] Mathôt, S., Schreij, D., & Theeuwes, J. (2012). OpenSesame: An open-source, graphical experiment builder for the social sciences. *Behavior Research Methods*, *44*(2), 314–324. 10.3758/s13428-011-0168-7 22083660 10.3758/s13428-011-0168-7 PMC3356517

[CR51] Mazerolle, M., Régner, I., Morisset, P., Rigalleau, F., & Huguet, P. (2012). Stereotype threat strengthens automatic recall and undermines controlled processes in older adults. *Psychological Science*, *23*(7), 723–727. 10.1177/0956797612437607 22609539 10.1177/0956797612437607

[CR52] McDonald, R. P. (1999). *Test Theory: A unified treatment* (1st ed.). Psychology.

[CR53] Monteiro, F., Nascimento, L. B., Leitão, J., Santos, E. J., Rodrigues, P., Santos, I. M., Simões, F., & Nascimento, C. S. (2024). OpenWMB: An open-source and automated working memory task battery for OpenSesame. *Behavior Research Methods*10.3758/s13428-024-02397-1 38575775 10.3758/s13428-024-02397-1 PMC11362385

[CR54] Muthén, L. K., & Muthén, B. O. (2002). How to use a Monte Carlo study to decide on sample size and determine power. *Structural Equation Modeling*, *9*(4), 599–620. 10.1207/S15328007SEM0904_8

[CR55] Oberauer, K., Süß, H. M., Wilhelm, O., & Wittman, W. W. (2003). The multiple faces of working memory: Storage, processing, supervision, and coordination. *Intelligence*, *31*(2), 167–193. 10.1016/S0160-2896(02)00115-0

[CR56] Oswald, F. L., McAbee, S. T., Redick, T. S., & Hambrick, D. Z. (2015). The development of a short domain-general measure of working memory capacity. *Behavior Research Methods*, *47*(4), 1343–1355. 10.3758/s13428-014-0543-2 25479734 10.3758/s13428-014-0543-2

[CR57] Pinheiro, J., Bates, D., DebRoy, S., Sarkar, D., Heisterkamp, S., Van Willigen, B., Ranke, J., & R Core Team. (2024). &. nlme: Linear and nonlinear mixed effects models. R package version 3.1–166. Retrieved from: https://cran.r-project.org/web/packages/nlme/index.html

[CR58] Pornprasertmanit, S., Miller, P., Schoemann, A., & Jorgensen, T. (2022). simsem: SIMulated structural equation modeling. R package version 0.5–16. Retrieved from: https://cran.r-project.org/web/packages/simsem/index.html

[CR59] Quinette, P., Guillery-Girard, B., Noël, A., de la Sayette, V., Viader, F., Desgranges, B., & Eustache, F. (2006). The relationship between working memory and episodic memory disorders in transient global amnesia. *Neuropsychologia*, *44*(12), 25082519. 10.1016/j.neuropsychologia.2006.03.031 10.1016/j.neuropsychologia.2006.03.031 16697428

[CR60] R Core Team. (2013). *R: A language and environment for statistical computing*. R Foundation for Statistical Computing. http://www.r-project.org/index.html

[CR61] Raven, J., Raven, J. C., & Court, J. H. (1998). *Manual for Raven’s Progressive Matrices and Vocabulary Scales. Section 4: the Advanced Progressive Matrices*. Oxford Psychologists.

[CR62] Redick, T. S., Broadway, J. M., Meier, M. E., Kuriakose, P. S., Unsworth, N., Kane, M. J., & Engle, R. W. (2012). Measuring working memory capacity with automated complex span tasks. *European Journal of Psychological Assessment*, *28*(3), 164–171. 10.1027/1015-5759/a000123

[CR63] Revelle, W. (2022). psych: Procedures for psychological, psychometric, and personality research. R package version 0.5.16. Retrieved from: https://cran.r-project.org/web/packages/psych/index.html

[CR64] Rey-Mermet, A., Gade, M., Souza, A. S., von Bastian, C. C., & Oberauer, K. (2019). Is executive control related to working memory capacity and fluid intelligence? *Journal of Experimental Psychology: General*, *148*(8), 1335–1372. 10.1037/xge0000593 30958017 10.1037/xge0000593

[CR65] Rimm-Kaufman, S. E., Baroody, A. E., Larsen, R. A., Curby, T. W., & Abry, T. (2015). To what extent do teacher-student interaction quality and student gender contribute to fifth graders’ engagement in mathematics learning? *Journal of Educational Psychology*, *107*(1), 170–185. 10.1037/a0037252

[CR66] Robitzsch, A., & Frund, S. (2022). miceadds: Some additional multiple imputation functions, especially for ‘mice’. *R Package Version* 3.17-44. https://cran.r-project.org/web/packages/miceadds/index.html

[CR67] Romeu, J. L., & Ozturk, A. (1993). A comparative study of goodness-of-fit tests for multivariate normality. *Journal of Multivariate Analysis*, *46*(2), 309–334. 10.1006/jmva.1993.1063

[CR68] Rosseel, Y. (2012). Lavaan: An R package for structural equation modeling. *Journal of Statistical Software*, *48*(2), 1–36. 10.18637/jss.v048.i02

[CR69] Rubin, D. B. (1987). *Multiple imputation for nonresponse in surveys* (1st ed.). Wiley.

[CR70] Salthouse, T. A., Babcock, R. L., & Shaw, R. I. (1991). Effects of adult age on structural and operational capacities in working memory. *Psychology and Aging*, *6*(1), 118–127. 10.1037/0882-7974.6.1.118 2029360 10.1037//0882-7974.6.1.118

[CR71] Schmidt, C., Collette, F., Reichert, C. F., Maire, M., Vandewalle, G., Peigneux, P., & Cajochen, C. (2015). Pushing the limits: Chronotype and time of day modulate working memory-dependent cerebral activity. *Frontiers in Neurology,**6*, 199. 10.3389/fneur.2015.00199 26441819 10.3389/fneur.2015.00199 PMC4585243

[CR72] Schmiedek, F., Hildebrandt, A., Lövdén, M., Wilhelm, O., & Lindenberger, U. (2009). Complex span versus updating tasks of working memory: The gap is not that deep. *Journal of Experimental Psychology: Learning Memory and Cognition*, *35*(4), 1089–1096. 10.1037/a0015730 19586272 10.1037/a0015730

[CR73] Schmiedek, F., Lövdén, M., & Lindenberger, U. (2014). A task is a task is a task: Putting complex span, n-back, and other working memory indicators in psychometric context. *Frontiers in Psychology*, *5*, 1475. 10.3389/fpsyg.2014.01475 25566149 10.3389/fpsyg.2014.01475 PMC4274887

[CR74] Schrepp, M. (1999). An empirical test of a process model for letter series completion problems. In D. Albert, & L. Lukas (Eds.), *Knowledge spaces: Theories, empirical research, and applications* (1st ed., pp. 133–154). Lawrence Erlbaum Associates.

[CR75] Shelton, J. T., Elliott, E. M., Hill, B. D., Calamia, M. R., & Gouvier, W. D. (2009). A comparison of laboratory and clinical working memory tests and their prediction of fluid intelligence. *Intelligence*, *37*(3), 283–293. 10.1016/j.intell.2008.11.005 20161647 10.1016/j.intell.2008.11.005 PMC2818304

[CR76] Shipstead, Z., Redick, T. S., & Engle, R. W. (2012). Is working memory training effective? *Psychological Bulletin*, *138*(4), 628–654. 10.1037/a0027473 22409508 10.1037/a0027473

[CR77] Simon, H. A., & Kotovsky, K. (1963). Human acquisition of concepts for sequential patterns. *Psychological Review*, *70*(6), 534–546. 10.1037/h0043901 14057302 10.1037/h0043901

[CR78] Spearman, C. C. (1910). Correlation calculated from faulty data. *British Journal of Psychology*, *3*, 271–295. 10.1111/j.2044-8295.1910.tb00206.x

[CR79] Stanton, J. M., Sinar, E. F., Balzer, W. K., & Smith, P. C. (2002). Issues and strategies for reducing the length of self-report scales. *Personnel Psychology*, *55*(1), 167–194. 10.1111/j.1744-6570.2002.tb00108.x

[CR80] Stollery, B., & Christian, L. (2016). Glucose improves object-location binding in visual-spatial working memory. *Psychopharmacology,**233*(3), 529–547. 10.1007/s00213-015-4125-5 26576942 10.1007/s00213-015-4125-5 PMC4710657

[CR81] Tavakol, M., & Dennick, R. (2011). Making sense of Cronbach’s alpha. *International Journal of Medical Education*, *2*, 53–55. 10.5116/ijme.4dfb.8dfd 28029643 10.5116/ijme.4dfb.8dfd PMC4205511

[CR82] Thurstone, L. L. (1938). *Primary mental abilities*. University of Chicago Press.

[CR83] Turner, M. L., & Engle, R. W. (1989). Is working memory capacity task dependent? *Journal of Memory and Language*, *28*(2), 127–154. 10.1016/0749-596X(89)90040-5

[CR84] Unsworth, N., & Engle, R. W. (2005). Working memory capacity and fluid abilities: Examining the correlation between operation span and Raven. *Intelligence*, *33*(1), 67–81. 10.1016/j.intell.2004.08.003

[CR85] Unsworth, N., Heitz, R. P., Schrock, J. C., & Engle, R. W. (2005). An automated version of the operation span task. *Behavior Research Methods*, *37*, 498–505. https://doi.org/10.3758 /BF03192720 16405146 10.3758/bf03192720

[CR86] Unsworth, N., Brewer, G. A., & Spillers, G. J. (2009a). There’s more to the working memory capacity-fluid intelligence relationship than just secondary memory. *Psychonomic Bulletin and Review*, *16*(5), 931–937. 10.3758/PBR.16.5.931 19815801 10.3758/PBR.16.5.931

[CR87] Unsworth, N., Redick, T. S., Heitz, R. P., Broadway, J. M., & Engle, R. W. (2009b). Complex working memory span tasks and higher-order cognition: A latent-variable analysis of the relationship between processing and storage. *Memory (Hove, England)*, *17*(6), 635–654. 10.1080/09658210902998047 19536691 10.1080/09658210902998047

[CR88] Van Buuren, S., & Groothuis-Oudshoorn, K. (2011). Mice: Multivariate imputation by chained equations in R. *Journal of Statistical Software,**45*, 1–67. 10.18637/jss.v045.i03

[CR89] Van Eekelen, A. P., & Kerkhof, G. A. (2003). No interference of task complexity with circadian rhythmicity in a constant routine protocol. *Ergonomics*, *46*(15), 1578–1593. 10.1080/0014013031000121598 14668176 10.1080/0014013031000121598

[CR90] Van Poucke, S., Zhang, Z., Roest, M., Vukicevic, M., Beran, M., Lauwereins, B., Zheng, M. H., Henskens, Y., Lancé, M., & Marcus, A. (2016). Normalization methods in time series of platelet function assays: A SQUIRE compliant study. *Medicine*, *95*(28), e4188. 10.1097/MD.0000000000004188 27428217 10.1097/MD.0000000000004188 PMC4956811

[CR91] Waris, O., Soveri, A., Ahti, M., Hoffing, R. C., Ventus, D., Jaeggi, S. M., Seitz, A. R., & Laine, M. (2017). A latent factor analysis of working memory measures using large-scale data. *Frontiers in Psychology*, *8*, 1062. 10.3389/fpsyg.2017.01062 28701976 10.3389/fpsyg.2017.01062 PMC5487690

[CR92] Waris, O., Jylkkä, J., Fellman, D., & Laine, M. (2021). Spontaneous strategy use during a working memory updating task. *Acta Psychologica,**212*, 103211. 10.1016/j.actpsy.2020.103211 33220613 10.1016/j.actpsy.2020.103211

[CR93] Wilhelm, O., Hildebrandt, A., & Oberauer, K. (2013). What is working memory capacity, and how can we measure it? *Frontiers in Psychology*, *4*, 433. 10.3389/fpsyg.2013.00433 23898309 10.3389/fpsyg.2013.00433 PMC3721021

[CR94] Wolf, E. J., Harrington, K. M., Clark, S. L., & Miller, M. W. (2013). Sample size requirements for structural equation models: An evaluation of power, bias, and solution propriety. *Educational and Psychological Measurement*, *73*(6), 913–934. https://doi.org/10.1177/ 0013164413495237 10.1177/0013164413495237 PMC4334479 25705052

